# Serological evidence of substantial respiratory syncytial virus infection burden among older adults residing in Swedish long-term care facilities

**DOI:** 10.1186/s12916-026-04700-7

**Published:** 2026-02-24

**Authors:** Preeti Moar, Christoffer Granvik, Kim Blom, Erick Bermúdez-Méndez, Florian Gegenfurtner, Julia Wigren Byström, Peter Fjällström, Mikael Åberg, Johan Normark, Karin Loré, Anders F. Johansson, Mattias N. E. Forsell

**Affiliations:** 1 Department of Clinical Microbiology, Umeå University, 90187 Umeå, Sweden; 2 Public Health Agency of Sweden, Solna, Sweden; 3 Division of Immunology and Respiratory Medicine, Department of Medicine Solna, Karolinska Institutet and Karolinska University Hospital, Stockholm, Sweden; 4 Center for Molecular Medicine, Karolinska Institutet, Stockholm, Sweden; 5 Department of Medical Sciences, Uppsala University, Uppsala, Sweden; 6 Science for Life Laboratory, Uppsala University, Uppsala, Sweden; 7 Science for Life Laboratory, Department of Clinical Microbiology, Umeå University, Umeå, Sweden

**Keywords:** RSV, Influenza, SARS-CoV-2, Vaccination, Immune monitoring, Vulnerable/ high risk population, Population immunity, Longitudinal sampling, Risk factors, Mortality

## Abstract

**Background:**

Older adults (> 65 years) residing in long-term care facilities (LTCFs) are at elevated risk of severe outcomes from respiratory infections. Infections often remain undetected or present atypically in this population, leading to underdiagnosis. Our study aimed to estimate the respiratory virus infection burden, independent of symptom presentation, among older adults in Swedish LTCFs in the post-pandemic period (2021–2024).

**Methods:**

We leveraged capillary blood samples and coupled national registry data from 1622 LTCF residents (median age = 87). A multiplex platform was used to quantify antigen-specific IgG and IgM responses to RSV (pre-/post-F, strain A-specific G-protein), influenza-A (H1N1 and H3N2 HA), influenza-B (HA) and SARS-CoV-2 (spike). Linear mixed-effects models were used to demonstrate the dynamics of antibody levels over time, adjusted for age, sex and comorbidities.

**Results:**

RSV-specific antibody responses peaked in spring 2022 (*p* < 0.001), suggesting an impact of relaxed COVID-19-related restrictions on RSV exposure at LTCFs. RSV-specific antibodies subsequently declined over time until an increase during autumn 2023 (*p* < 0.001). Geographic variation in pre-F antibody levels suggested localised RSV outbreaks. The total estimated RSV burden at LTCFs was markedly higher than official reports of the Swedish Public Health Agency. Influenza antibody dynamics reflected seasonal trends and were strongly influenced by annual vaccination. A random forest classifier incorporating serological profiles with demographics, location and comorbidities significantly outperformed a model without serological data (AUC-ROC = 0.67 vs. 0.58), although discriminatory performance remained modest. Higher levels of RSV pre-F antibodies in autumn 2021 were associated with increased one-year mortality in logistic regression (OR = 1.43, *p* = 0.024). Exploratory survival analysis indicated a trend that elevated levels of RSV pre-F antibodies during low population immunity may confer a transiently elevated early hazard of death, although this did not reach statistical significance (HR = 4.50, *p* = 0.087).

**Conclusions:**

We observed substantial respiratory virus circulation among older adults in Swedish LTCFs and show that RSV burden is under-reported. The results highlight a need for further research into the role of RSV pre-F antibody levels in preventing severe outcomes, potentially via vaccination of LTCF residents. Our scalable serological surveillance system is a valuable approach to detect respiratory infections in LTCFs, independent of symptom presentation or healthcare-seeking behaviour.

**Supplementary Information:**

The online version contains supplementary material available at 10.1186/s12916-026-04700-7.

## Background

Respiratory syncytial virus (RSV), influenza viruses and severe acute respiratory syndrome coronavirus 2 (SARS-CoV-2) are major causes of respiratory morbidity and mortality in older adults and individuals with chronic conditions [1, 2]. During the COVID-19 pandemic, non-pharmaceutical interventions markedly suppressed RSV and influenza circulation [3, 4], followed by the resurgence of both viruses in 2021–2022 as restrictions were relaxed [5–7]. Older adults over 65 years of age residing in long-term care facilities (LTCFs) are especially vulnerable to severe respiratory infections due to age-related immune decline and high prevalence of comorbidities [8], yet seroepidemiologic data on RSV and influenza in this group remain scarce.

RSV is a ubiquitous pathogen that infects nearly all individuals at a young age, and re-infections may occur throughout life [9, 10]. While it is well recognised as a leading cause of bronchiolitis and pneumonia in infants, RSV also causes significant disease burden in older adults, especially those living in LTCFs, conferring high rates of hospitalisation and mortality [11–13]. Despite this, RSV prevention has traditionally focused on pediatric populations. Recent advances, including the licensure of RSV vaccines for older adults, have prompted new vaccination recommendations [14, 15]. The vaccine has been shown to reduce the incidence of hospitalisation for RSV-related respiratory tract diseases among adults over 60 years of age [16]. In Sweden, LTCFs are prioritised for biannual SARS-CoV-2 and annual influenza vaccination in cost-free vaccination programmes. In 2023, the Public Health Agency of Sweden (PHAS) recommended RSV vaccination for adults over 75 years and those over 60 years with specific underlying conditions. However, the vaccine is not subsidised and national data on uptake among older adults are currently lacking in Sweden.

The highly conserved RSV fusion (F) glycoprotein in its pre-fusion (pre-F) conformation is the primary target of broadly neutralising antibodies, whereas the attachment glycoprotein (G) is more variable and elicits weaker, strain-specific responses [17–19]. Protection against RSV is afforded mainly by neutralising antibodies [20]. In contrast, immunity to influenza is shaped by prior exposure and vaccination and varies annually due to antigenic drift and shift, necessitating regular vaccine updates [21, 22]. However, the durability and temporal dynamics of RSV- and influenza-specific humoral immunity in LTCF residents remain poorly understood, particularly in the post-pandemic period when viral circulation patterns and host susceptibility were altered.

To address this gap, we leveraged an established national biomonitoring platform involving quarterly capillary blood sampling and registry linkage to longitudinally assess antibody dynamics to RSV, influenza and SARS-CoV-2 among LTCF residents in Sweden from 2021 to 2024. We have previously used this platform to assess vaccine-induced protection against COVID-19 [23]. Here, we report population-level exposure to respiratory infections, temporal and regional patterns of RSV antigen-specific immunity, influenza subtype-specific immune responses to infection and vaccination, and SARS-CoV-2 vaccine-induced antibody durability. Our findings provide essential insight into the burden of respiratory virus exposure in Swedish LTCFs and demonstrate the value of longitudinal serological surveillance. Collectively, these and complementary data may inform estimates of infection burden and guide future vaccination strategies in this population.

## Methods

### Study design and population

**Fig. 1:**
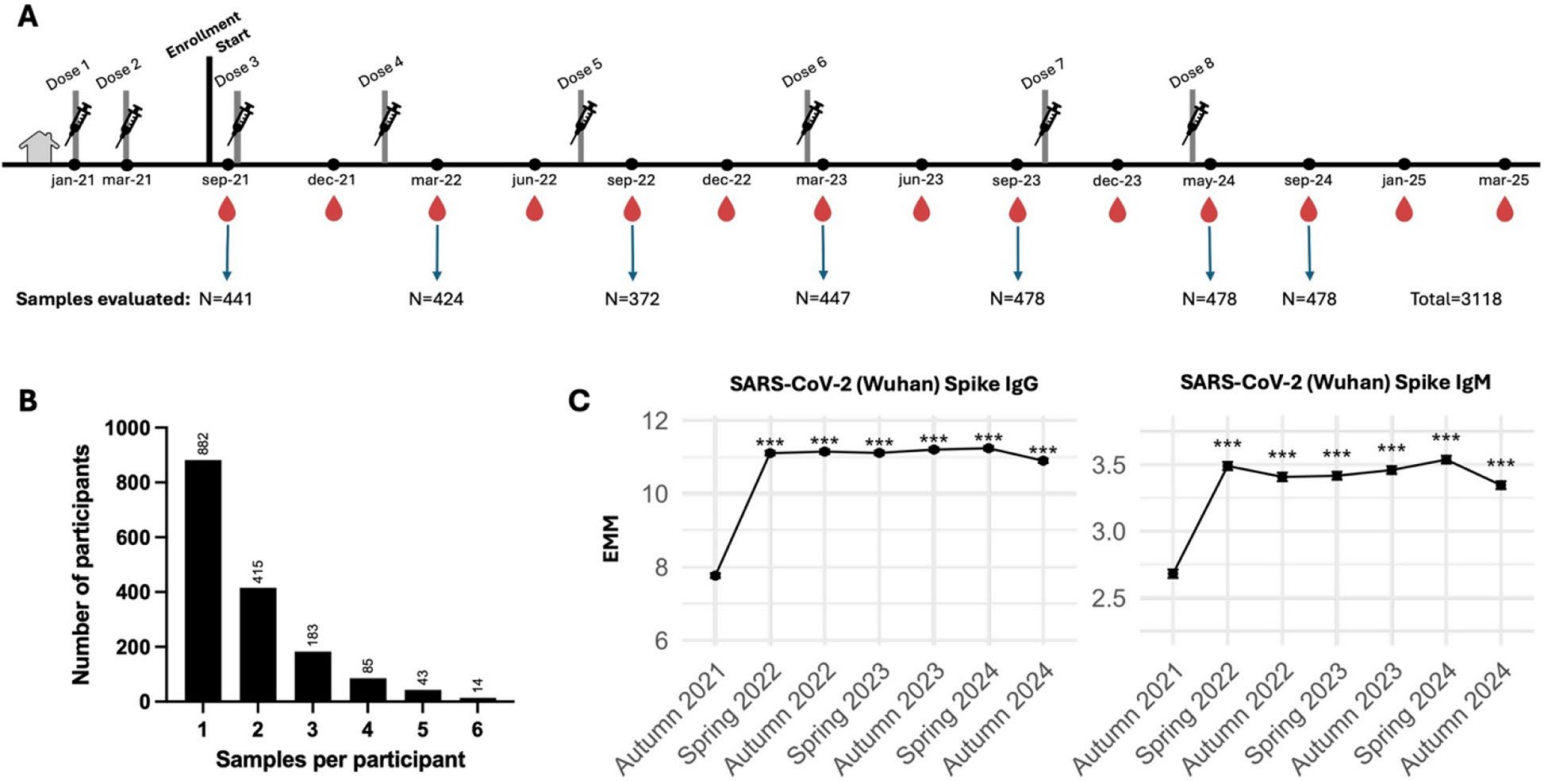
Study design and SARS-CoV-2-specific humoral responses following vaccination.** A** Sampling strategy indicating the number of samples assessed from each sampling period and the total number of tested samples. The blood drop indicates routine capillary blood sample collection and doses represent COVID-19 vaccination doses given to all participants in the cohort. **B** Frequency distribution of longitudinal samples evaluated per participant. **C** Estimated marginal means with 95% CI from LMMs, adjusted for age, sex, batch, and CCIw. Statistical significance is shown relative to autumn 2021; ***p < 0.001

Older adults (> 65 years) residing in LTCFs in five regions of Sweden (Jämtland-Härjedalen, Örebro, Skåne, Stockholm and Västerbotten) were enrolled in an open cohort longitudinal observational study to investigate correlates of protection against respiratory viral infections. Samples were collected every 3 months until 2024 and every 4 months thereafter. All study participants had received two doses of the COVID-19 vaccine before the first sampling period. We utilised capillary blood samples collected from this previously established cohort to monitor the seroprevalence of other respiratory infections caused by RSV and influenza viruses. Seven sampling periods from autumn 2021 to autumn 2024 were included with an average of 445 samples from each sampling period. This ensured longitudinal follow-up of a proportion of the individuals throughout the study period. Participant characteristics and sampling strategy are summarised in Table 1 and Fig. 1A. Serology data from individuals were linked with registry data using the personal identity number given to everyone registered in Sweden. The study was approved by the Swedish Ethical Review Board (decision 2021–00055 with amendments) and conducted with informed consent and respect to human dignity in accordance with the Ethical Review Act of Sweden.

**Table 1:** Participant characteristics

	**Overall**, *N* = 1622^*1*^	**Female**, *N* = 1062^*1*^	**Male**, *N* = 560^*1*^	***p*-value**^*2*^
Age	87 (81, 92)	88 (83, 93)	84 (79, 89)	< 0.001
Jämtland	256 (15.8%)	157 (14.8%)	99 (17.7%)	-
Örebro	387 (23.9%)	260 (24.5%)	127 (22.7%)	-
Skåne	193 (11.9%)	129 (12.1%)	64 (11.4%)	-
Stockholm	121 (7.5%)	79 (7.4%)	42 (7.5%)	-
Västerbotten	665 (41%)	437 (41.1%)	228 (40.7%)	-
Myocardial infarction	211 (13%)	103 (9.7%)	108 (19%)	< 0.001
Congestive heart failure	268 (17%)	153 (14%)	115 (21%)	0.002
Peripheral vascular disease	119 (7.3%)	64 (6.0%)	55 (9.8%)	0.005
Cerebrovascular disease	452 (28%)	256 (24%)	196 (35%)	< 0.001
Chronic obstructive pulmonary disease	98 (6.0%)	66 (6.2%)	32 (5.7%)	0.688
Chronic other pulmonary disease	118 (7.3%)	74 (7.0%)	44 (7.9%)	0.512
Rheumatic disease	118 (7.3%)	89 (8.4%)	29 (5.2%)	0.018
Dementia	839 (52%)	579 (55%)	260 (46%)	0.002
Hemiplegia	153 (9.4%)	81 (7.6%)	72 (13%)	< 0.001
Diabetes without chronic complication	274 (17%)	157 (15%)	117 (21%)	0.002
Diabetes with chronic complication	113 (7.0%)	62 (5.8%)	51 (9.1%)	0.014
Renal disease	89 (5.5%)	40 (3.8%)	49 (8.8%)	< 0.001
Mild liver disease	14 (0.9%)	6 (0.6%)	8 (1.4%)	0.091
Ascites	6 (0.4%)	5 (0.5%)	1 (0.2%)	0.671
Severe liver disease	2 (0.1%)	1 (< 0.1%)	1 (0.2%)	> 0.999
Peptic ulcer disease	73 (4.5%)	40 (3.8%)	33 (5.9%)	0.050
Malignancy	226 (14%)	122 (11%)	104 (19%)	< 0.001
Metastatic solid tumor	30 (1.8%)	16 (1.5%)	14 (2.5%)	0.158
Aids	1 (< 0.1%)	0 (0%)	1 (0.2%)	0.345
CCIunw	2.00 (1.00, 3.00)	2.00 (1.00, 2.00)	2.00 (1.00, 3.00)	< 0.001
CCIw	2.00 (1.00, 3.00)	2.00 (1.00, 3.00)	3.00 (1.00, 4.00)	< 0.001

Abbreviations: *N*, number of participants; *IQR*, interquartile range

^*1*^Median (IQR); N (%)

^*2*^Wilcoxon rank sum test; Pearson’s Chi-squared test; Fisher’s exact test

### Sampling technique

Capillary blood samples were collected in the form of dried blood spots (DBS) as previously described [24] with few modifications. Briefly, individual capillary blood sampling kits were sent to study participants via postal services and sampling was conducted by finger pricking by the individuals themselves or assisted by personnel at the LTCFs. These samples were then sent back to the laboratory where 10 µL capillary blood was eluted using 100 µL Phosphate Buffered Saline with 0.05% Tween-20 (PBS-T) containing protease inhibitor cocktail (Cat# 4693116,001, Roche).

### Expression and purification of antigens

The SARS-CoV-2 (Wuhan strain) spike protein was produced using the Freestyle MAX 293 Expression System (Thermo Fisher Scientific) and has been previously described [24]. RSV strain A–specific glycoprotein (G) and influenza strain-specific hemagglutinin (HA) proteins were procured from commercial vendors (Sino Biological). Antigen details and catalog information are summarised in Additional file 1: S1. Recombinant RSV strain A–specific pre-F (SC-DM) [25] and post-F (RSV-F dFP) [17] proteins were expressed in Expi293F cells (Gibco, CVCL_D615), a suspension-adapted human embryonic kidney cell line. Codon-optimised gene sequences for RSV pre-F and post-F, each containing C-terminal 8 × His and Avi tags, were synthesised and cloned into CMVR_HA1 plasmids by GenScript (Rijswijk, The Netherlands). Transient transfection was performed at a viable cell density of 3 × 10^6^ cells/mL using 1 µg plasmid DNA/mL and 3 µg/mL of PEI MAX MW 40,000 (Polysciences). For RSV post-F expression, a furin plasmid was co-transfected at 1:4 furin to post-F plasmid ratio. Culture supernatants were harvested four days post-transfection, clarified by centrifugation and filtered through a 0.22 µm vacuum-driven sterile filter. Supernatants were then pH- and salt-stabilised to a final concentration of 10 mM PBS pH 7.4. Protein purification involved a two-step process: affinity-based capture followed by size exclusion chromatography. Proteins were bound to Ni^+2^-Sepharose excel resin (Cytiva) by incubation at 4 °C for 1 h with head-to-heel rotation and eluted via gravity flow using 500 mM imidazole in PBS. Eluates were concentrated using Amicon Ultra centrifugal filters (Millipore) and further purified on a Superdex 200 Increase 10/300 GL column (Cytiva) equilibrated in 10 mM PBS pH 7.4.

### Quantification of antigen-specific antibodies from capillary blood

We developed a bead-based multiplex serological assay based on Luminex technology. The viral antigens from SARS-CoV-2 (Wuhan strain, spike protein), RSV (pre-F, post-F and G protein) and influenza (InfA H1N1, H3N2 and InfB HAs) were coupled to magnetic MagPlex 6.5 µm COOH-microspheres (Luminex Corporation) following the manufacturer’s protocols (xMAP Cookbook, 5th edition). Color-coded microspheres, also referred to as beads, are internally dyed with different proportions of red and infrared fluorophores that correspond to a distinct spectral signature or bead region. Each antigen was coupled to a unique bead region to enable multiplexing. Antigen concentrations used for coupling are summarised in Additional file 1: S1. The antigen-coupled microspheres were added to a 96-well flat-bottom black plate (Cat# 237108, Thermo Scientific) at a concentration of 500 microspheres of each set/well in a total volume of 50 µL PBS-T containing 0.1% bovine serum albumin and 0.05% sodium azide (PBS-TBN). Capillary blood samples diluted 1/200 in a total volume of 50 µL/well were added to the antigen-coupled microspheres mixture and incubated at room temperature (RT) for 1 h on a microplate shaker (Thermo Scientific) at 800 rpm protected from light. A pooled serum control, composed of six samples with antibodies against all seven assay antigens, was included in duplicate on each plate. Duplicate blank wells containing only antigen-coupled microspheres in PBS-TBN were also added to monitor background. After the first incubation, plates were washed thrice with 200 µL/well of PBS-TBN, using an automated plate washer with magnetic adaptor (BioTek 406 with BioStack 3WR, Agilent Technologies). Further, 50 µL of 1 µg/mL fluorescently labelled isotype-specific secondary antibodies: anti-human IgG Fc-PE (Cat# 9040–09, SouthernBiotech) and anti-human IgM-BV421 (Cat# 562618, BD Biosciences) diluted in PBS-TBN were added to all wells and incubated at RT for 1 h at 800 rpm protected from light. Plates were washed three times and beads were resuspended in 60 µL of PBS-TBN for analysing on the Intelliflex DR-SE (Luminex Corporation) with 40 µL of acquisition volume per well, doublet discriminator gate set at 7000–20,000, and dual reporter settings. The Intelliflex dual reporter system utilises two reporter lasers: 532 nm (primary reporter-RP1) and 405 nm (secondary reporter-RP2) and has the benefit of a broad dynamic range (≥ 5.5 logs for RP1 and ≥ 4.5 logs for RP2), enabling the detection of antigen-specific IgG and IgM at the same time in each sample well. The outcome was median fluorescence intensity (MFI) from 50 microspheres. Raw data was exported as a CSV file using the in-built xPONENT software.

### Comorbidity assessment

Comorbidity burden was assessed using the Charlson Comorbidity Index (CCI), widely used as a summary measure of comorbidity in epidemiological research. For this study, we applied a version of the CCI specifically adapted for Swedish register-based research [26]. CCI weighted score (CCIw) was included as a continuous covariate in the statistical models.

### Linear mixed-effects models

Linear mixed-effects models (LMMs) were used to assess associations between antibody levels, demographics and clinical variables, accounting for the repeated measures at different time-points. Participant ID was included as a random effect, antibody levels as a dependent variable, represented by log-transformed MFI from the multiplex serology assay, while fixed effects included age, sex, batch and CCIw. No imputation was applied. Models were fitted using the *lme4* package in R (version 4.2.2). Estimated marginal means and pairwise comparisons were computed using the *emmeans* package, with p-values adjusted for multiple comparisons using Tukey’s method.

### Infection burden

In the absence of a validated serological threshold for RSV and influenza infections in older adults with pre-existing immunity, we used fold increases in antibody levels between consecutive sampling periods as a proxy for infection or viral exposure. Specifically, we evaluated 1.5-, 2- and 4-fold increases in RSV pre-F antibody levels, informed by previous studies: 1.5-fold increase indicative of asymptomatic or subclinical exposure [27, 28], 2-fold increase that most likely reflects infection in the context of pre-existing immunity [29–31], and 4-fold increase, which is a clinically accepted seroconversion threshold [31, 32]. We use the terms “exposure” and “infection” interchangeably to indicate evidence of a recent immunologic response to RSV or influenza. The term “infection burden” refers to the proportion of individuals meeting one of these fold-rise criteria at the defined sampling periods.

### Random forest

To correct for systematic technical variation, antibody values were batch-normalised via mean-centering. Subsequently, group-wise mean-centering was applied across defined sampling seasons to account for temporal trends in antibody levels since many participants did not have more than one sample. For each participant, antibody responses were averaged across available time-points, and Principal Component Analysis (PCA) was performed separately on antibody profiles for RSV, influenza and SARS-CoV-2. Participants were then classified into two groups, alive or deceased, during the study period. This classification was based on the presence or absence of a recorded death date in the national tax registry database. Two random forest classification models were developed using the *ranger* package in R (version 4.2.2), each consisting of 1000 decision trees. Variable importance was evaluated using a permutation-based approach. The first model included only demographic and clinical covariates: age, gender, CCIw and postal code. The second model incorporated these covariates along with six principal components: first two principal components (PC1 and PC2) from each virus-specific group. The dataset was randomly split into training (70%) and testing (30%) subsets using stratified sampling. No resampling or weighting was applied. Model performance was evaluated on the test set using area under the receiver operating characteristic curve (AUC-ROC). Variable importance was visualised via permutation scores. ROC curves were generated for both models. The difference in AUC-ROC between the covariate-only and combined model was formally tested using DeLong's test and a bootstrap method (2000 replicates). Calibration of predicted probabilities from the combined model was assessed using bootstrap resampling (1000 replicates). Both apparent and bias-corrected calibration curves were generated to evaluate agreement between predicted and observed mortality at the group level. The Brier score was computed as the mean squared error between predicted probabilities and observed outcomes to assess overall probability accuracy.

### Survival analysis

Participant follow-up time was calculated from September 29, 2021, until death or administrative censoring on December 17, 2024. For participants with multiple measurements, each observation interval was defined by the sampling date and ended at the earliest of the next sampling date, death, or censoring. Sampling date was defined preferentially from the recorded sampling date; if unavailable, the laboratory arrival date minus five days. A Cox proportional hazards model was fitted with start–stop time to estimate the association between standardised log-transformed measurements of pre-F-specific IgG and mortality risk, adjusting for age, sex, batch and CCIw, with clustering on participant ID to account for repeated measures. To assess potential time-varying effects, we additionally included an interaction term between log-transformed antibody levels and log(time). Hazard ratio (HR) with 95% confidence interval (CI) was derived from model coefficients, and time-varying HR was plotted across the follow-up period using log(time).

### Statistical analyses

Trends in antibody levels were analysed using lognormal ordinary analysis of variance (ANOVA) and LMMs. Dunnett’s multiple comparison tests and Tukey’s corrections were included as post-hoc analyses. Batch normalisation was performed separately for IgG and IgM for each viral antigen. Antibody levels are directly comparable over time within a given antigen and antibody class, and hence, all analyses focused on within-antigen temporal dynamics and relative changes rather than cross-antigen comparisons. Participants with missing covariates were few (< 0.5%) and were excluded from the study. Complete case analyses were performed. Missing sampling dates were imputed using proxy data (date of arrival to the laboratory minus median time for postal delivery; 5.8% of the samples). Technical failures in serology assay were handled by excluding the failed antibody measures (1.2%), but the participant was still included in other analyses. Data was processed and visualised using Microsoft Excel, GraphPad Prism version 10.5.0 and R versions 4.2.2 and 4.4.1.

## Results

### Participant characteristics

The study included 1622 LTCF residents from five Swedish regions, with a median age of 87 years (interquartile range 81–92 years). Number of participants from each region and demographics including comorbidities are summarised in Table 1 and Additional file 1: S2. Both metropolitan and less densely populated regions were included (Additional file 1: S2-A). There were more female participants represented from each region than males (Additional file 1: S2-B), and most of the older participants were females (Additional file 1: S2-C). Participants received up to eight doses of SARS-CoV-2 vaccine during the study period. A total of 3118 samples corresponding to spring and autumn seasons each year spanning 2021 to 2024 were evaluated for antigen-specific antibody levels using the multiplex serology assay (Fig. 1A). Most participants had one sample analysed from a single time-point, 142 individuals had samples analysed from four or more sampling periods and 57 individuals had samples analysed from five or more sampling periods (Fig. 1B). Baseline characteristics were largely comparable between those who contributed a single blood sample (*n* = 881) and those with two or more samples (*n* = 741) during the study (Additional file 1: S3). Antibody measurements using our multiplex bead-based assay correlated strongly with an alternative assay platform (Spearman *r* = 0.89, *p* < 0.0001; Additional file 1: S4-A) and paired serum samples (Spearman *r* = 0.99, *p* < 0.0001; Additional file 1: S4-B).

### Sustained SARS-CoV-2 spike-specific antibody responses following repeated vaccination

All study participants received a third dose of the COVID-19 vaccination in autumn 2021 and showed persistent spike-specific antibody responses throughout the subsequent sampling periods (Additional file 1: S5). IgG and IgM levels increased significantly from baseline, i.e., September 2021, and remained elevated consistently through to September 2024 (*p* < 0.001 for all comparisons vs. baseline). To account for the longitudinal measures, we used LMMs to confirm associations between antibody levels at different sampling periods corresponding to seasons (Additional file 1: S6). LMMs capture longitudinal changes in antibody levels compared to baseline, i.e., the first sampling period in autumn 2021, while accounting for population-level trends and individual variability. The estimated marginal means (EMM) were plotted in the form of line graphs for visualisation. Even after adjusting for age, sex, batch and CCIw, both SARS-CoV-2-specific IgG and IgM levels sustained significantly higher compared to autumn 2021 (Fig. 1C).

### Temporal and geographical antibody fluctuations suggest significant underreporting of RSV exposure in LTCFs

**Fig. 2:**
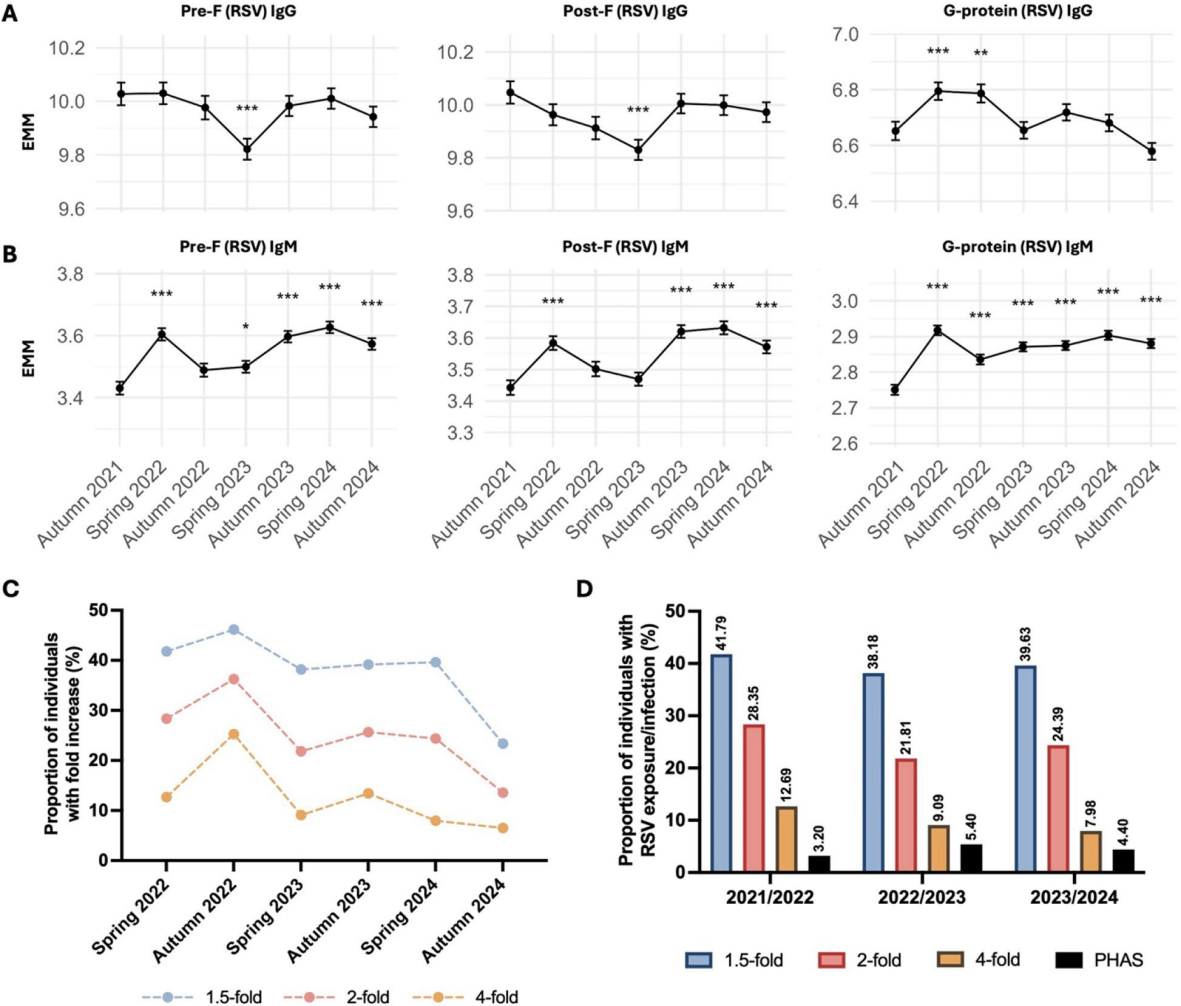
RSV-specific humoral responses and infection burden. RSV pre-F, post-F and G protein-specific (**A**) IgG and (**B**) IgM levels over time. Data points represent estimated marginal means (EMM) with 95% CI from LMMs, adjusted for age, sex, batch and CCIw. Statistical significance is relative to autumn 2021; *p < 0.05, **p < 0.01, ***p < 0.001. (C) Sensitivity analysis showing the proportion of individuals with at least 1.5-fold, 2-fold and 4-fold increase in RSV pre-F antibody levels between consecutive sampling periods during the study. Temporal trends are robust across thresholds, though absolute values vary, reflecting differing stringency in defining RSV exposure. (D) Proportion of individuals with RSV exposure/infection as determined by the fold increase in antibody levels using our multiplex serology assay compared to infection rates reported by PHAS during the corresponding RSV seasons

All individuals included in the analysis had RSV antibodies, and, since RSV vaccines were not included in the vaccination program during the study period, to the best of our knowledge, none of the participants received RSV vaccination. IgG and IgM responses against the RSV pre-F, post-F and G proteins were evaluated over the study period (Additional file 2: S7). The antibody levels in autumn 2021 during the COVID-19 restrictions were set as baseline. Changes from baseline were modeled using LMMs, adjusting for repeated measures, sex, age, batch and CCIw (Table 2). IgG levels to pre-F and post-F antigens remained relatively stable with a significant decrease in spring 2023 (*p* < 0.001; Fig. 2A). G-protein-specific IgG responses peaked in spring 2022 (*p* < 0.001) and subsequently declined through to autumn 2024 (Fig. 2A). In contrast, IgM responses against all three RSV antigens displayed dynamic patterns over time (Fig. 2B). Both F- and G-specific IgM levels increased significantly in spring 2022 (all *p* < 0.001) marking a significant increase in exposure to RSV that coincided with the end of COVID-19-related visiting restrictions at the LTCFs. F-specific IgM levels decreased in autumn 2022 but showed a significant increase later again in spring 2024 (all *p* < 0.001) suggesting significant RSV circulation in the LTCFs during winter. G-specific IgM levels persisted higher than baseline at all time-points indicating active infections in this population from spring 2022 to autumn 2024 (Fig. 2B). RSV pre-F and post-F IgG levels correlated significantly throughout the sampling time-points, whereas G-specific IgG levels showed a weaker association to F-specific IgG (Additional file 2: S8). Both F- and G-specific IgM levels showed a strong association with each other, most likely corresponding to RSV-A re-infections.

**Table 2:** Summary of LMMs to examine the relationship between RSV antigen-specific antibody levels at different sampling periods, adjusting for age, sex, batch and comorbidities

	Pre-F (RSV)		Post-F (RSV)		G protein (RSV)	
	IgG	IgM	IgG	IgM	IgG	IgM
Sampling period						
Spring 2022	0.0126	0.1739***	− 0.0843	0.1413***	0.1429***	0.1668***
	[− 0.09, 0.12]	[0.13, 0.22]	[− 0.18, 0.02]	[0.09, 0.19]	[0.07, 0.21]	[0.14, 0.20]
Autumn 2022	− 0.0653	0.0637*	− 0.1348*	0.0591*	0.1346***	0.0849***
	[− 0.18, 0.05]	[0.01, 0.11]	[− 0.24, − 0.03]	[0.00, 0.12]	[0.06, 0.21]	[0.05, 0.12]
Spring 2023	− 0.2008***	0.0689**	− 0.2171***	0.0268	0.0025	0.1205***
	[− 0.31, − 0.09]	[0.02, 0.12]	[− 0.32, − 0.12]	[− 0.03, 0.08]	[− 0.07, 0.07]	[0.09, 0.15]
Autumn 2023	− 0.0387	0.164***	− 0.0419	0.1779***	0.0667	0.1242***
	[− 0.14, 0.07]	[0.12, 0.21]	[− 0.14, 0.06]	[0.13, 0.23]	[0.00, 0.14]	[0.09, 0.16]
Spring 2024	− 0.0123	0.1951***	− 0.0482	0.1895***	0.0288	0.1529***
	[− 0.12, 0.09]	[0.15, 0.24]	[− 0.15, 0.05]	[0.14, 0.24]	[− 0.04, 0.10]	[0.12, 0.19]
Autumn 2024	− 0.0794	0.1403***	− 0.0746	0.1291***	− 0.0728*	0.1299***
	[− 0.19, 0.03]	[0.09, 0.19]	[− 0.18, 0.03]	[0.08, 0.18]	[− 0.14, 0.00]	[0.10, 0.16]
Age	0.0069*	0.0063***	0.0067*	0.0062***	0.006*	0.0014
	[0.0016, 0.0122]	[0.003, 0.009]	[0.0012, 0.0121]	[0.003, 0.009]	[0.001, 0.011]	[-0.001, 0.004]
Sex						
Male	0.208***	− 0.0331	0.1342**	− 0.0659*	0.1517***	− 0.0636***
	[0.12, 0.29]	[− 0.08, 0.02]	[0.05, 0.22]	[− 0.12, − 0.01]	[0.07, 0.24]	[− 0.10, − 0.03]
CCIw	− 0.0045	− 0.0035	0.0016	0.0016	− 0.0018	− 0.0009
	[− 0.02, 0.02]	[− 0.02, 0.01]	[− 0.02, 0.02]	[− 0.011, 0.014]	[− 0.022, 0.019]	[− 0.009, 0.007]

Estimate [95% CI]

*p < 0.05

**p < 0.01

***p-value < 0.001

To determine the infection burden of RSV in Swedish LTCFs, we quantified the proportion of individuals with an increase in RSV pre-F-specific antibodies compared to the previous sampling period. Only individuals who had samples available at two consecutive time-points were included in this analysis, and an increase in either IgG or IgM or both was counted as an infection. There is no consensus on a set threshold for fold increase, in the context of pre-existing immunity, to determine RSV re-infection/exposure. We determined exposure to RSV using three different thresholds for fold increase in antibody levels (Fig. 2C). Temporal trends were robust across thresholds, though absolute values varied. This increase in antibody levels was primarily infection-driven as the RSV vaccine was not included in the vaccination program for older adults in Sweden during the sampling periods included in the study. As per our data, the highest proportion of individuals were exposed to RSV in autumn 2022, following the end of COVID-19-related visiting restrictions in spring 2022 (Fig. 2C). RSV exposure declined in spring 2023, potentially due to acquired population immunity, leading to an increase in RSV infections in autumn 2023, building up population immunity, with a consequential decline in exposure to RSV in 2024 (Fig. 2C).

The proportion of individuals exposed to RSV as determined by our serology assay even with the most conservative threshold, i.e., 4-fold-increase, were higher than that reported by PHAS (Fig. 2D), implying that the RSV burden in LTCFs is higher than in the general population. However, there are key methodological differences between the RSV infections reported by PHAS and our infection burden estimates. Unlike the case-based PCR testing by PHAS, which captures symptomatic individuals seeking care, our cohort-based surveillance system relies on longitudinal serology data to estimate RSV exposure in unselected LTCF residents, enabling detection of both symptomatic and asymptomatic infections.

**Fig. 3:**
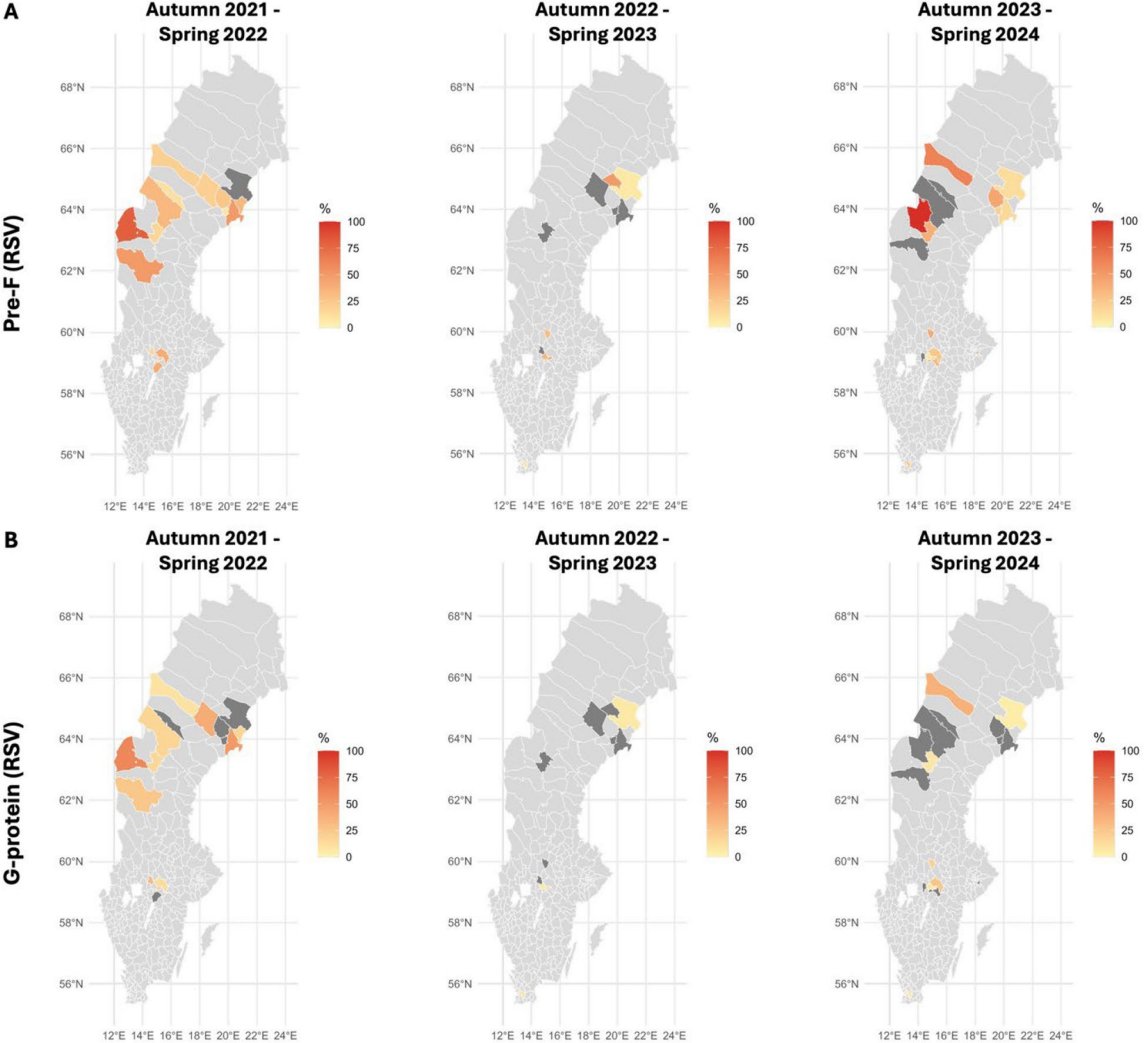
Spatial and temporal trends in RSV-specific antibody responses across Sweden. Infection rates across five regions of Sweden during the 2022, 2023 and 2024 RSV seasons. Heatmaps represent the proportion of individuals in spring with at least a 2-fold increase in **A** RSV pre-F- and **B** G-specific IgG or IgM, compared to the previous autumn sampling. Dark grey areas represent that none of the individuals from that location had a twofold increase in IgG or IgM levels

To assess spatial trends in RSV infections, we mapped the infection rates to geographical locations of the LTCFs across the five regions of Sweden (Fig. 3). The maps reflect infection rates for the RSV season, i.e., autumn to spring, for the three consecutive years. Skåne and Stockholm regions were underrepresented as there were few individuals with samples assessed from consecutive time-points. An increase in the levels of RSV antibodies was observed in spring 2022 in all five regions (Additional file 2: S9). We observed broad regional spread with high infection/exposure rates in regions Jämtland-Härjedalen and Västerbotten (Fig. 3), suggesting intense RSV circulation during the season immediately after the COVID-19-related restrictions were removed in Swedish LTCFs. In contrast, 2023 was marked by a reduction in RSV antibodies, indicative of a milder RSV season. Presumably as a consequence of waning immunity, there was RSV resurgence in 2024, particularly in the northern regions, for both pre-F and G-protein antigens. Interestingly, some locations with high F-specific exposure rates did not have increased G-specific antibodies, indicating an occurrence of strain B, as the G-protein used in the multiplex assay was derived from strain A.

### Seasonal influenza antibody dynamics reflect robust vaccine-induced responses

**Fig. 4:**
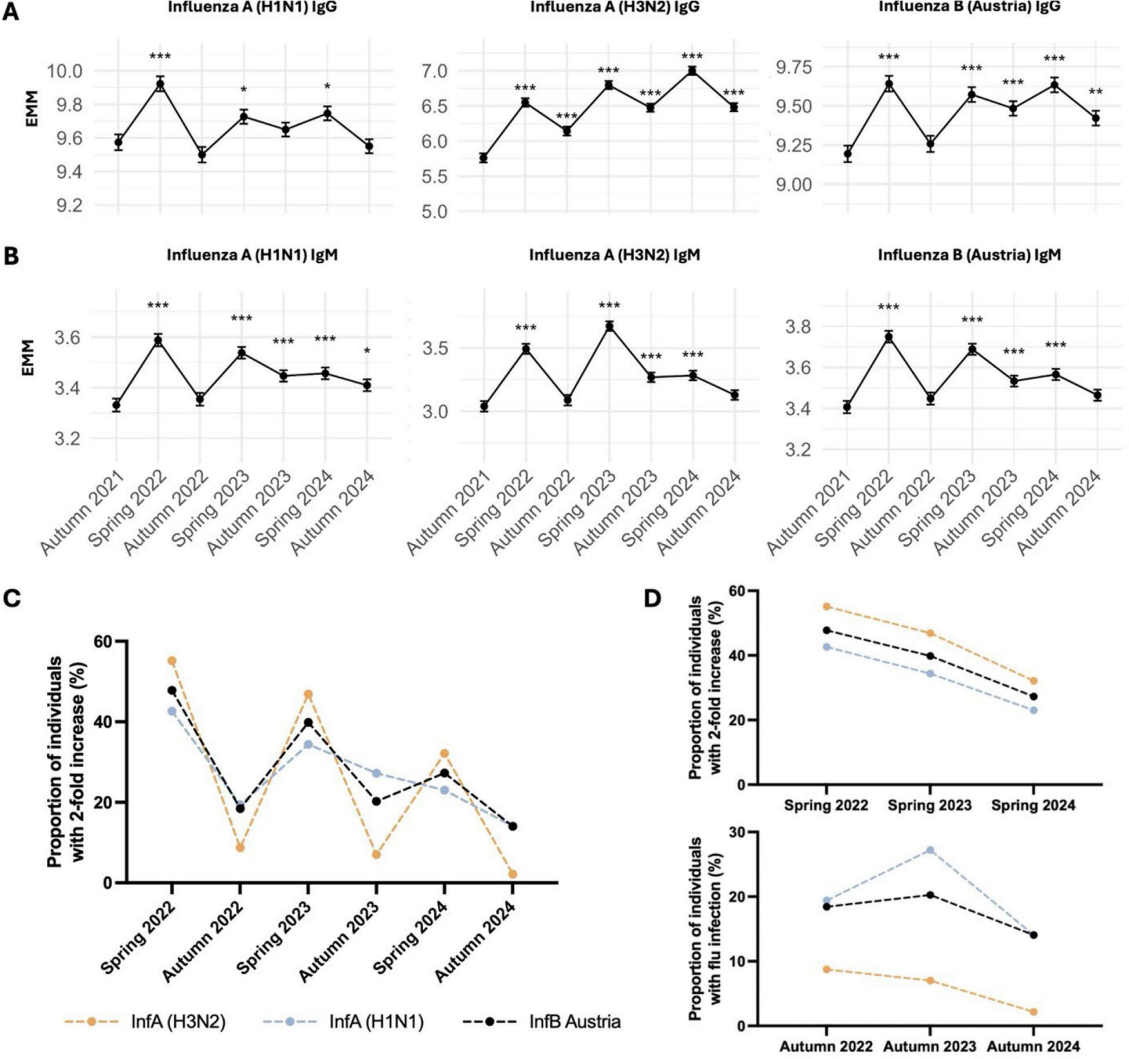
Influenza strain-specific humoral responses and effect of vaccination. Influenza strain-specific (**A**) IgG and (**B**) IgM levels over time. Data points represent estimated marginal means with 95% CI from LMMs, adjusted for age, sex, batch and CCIw. Statistical significance is shown relative to autumn 2021; *p < 0.05, **p < 0.01, ***p < 0.001. **C** Proportion of participants with at least 2-fold increase in either IgG or IgM since the last time-point across six sampling seasons. This change in antibody levels is primarily vaccination-driven, but infections cannot be excluded. **D** Proportion of participants showing an increase in antibody levels corresponding to the effects of vaccination (top panel) and influenza infections (bottom panel). The change in antibody levels in spring is primarily vaccination-driven as the vaccine is given in late autumn. The change in antibody levels in autumn, however, is primarily infection-driven

A majority of individuals in the Swedish LTCFs are given the influenza vaccine in late autumn which is a combination of the predicted circulating strains for that season. The influenza strains included in our assay corresponded to the strains used in the annual vaccine. To assess seasonal antibody responses to influenza strains (InfA H1N1, H3N2 and InfB) and the effect of vaccination, we quantified strain-specific IgG and IgM levels over seven sampling periods spanning autumn 2021–2024 (Additional file 2: S10). Changes from baseline were modeled using LMMs for repeated measures, adjusting for sex, age, batch and CCIw (Table 3). Unlike RSV, a strong seasonal trend was observed in the seroprevalence of influenza with a clear boost in IgG levels each spring (Fig. 4A), presumably as an effect of the autumn vaccination. There was also a significant increase in IgM levels each spring (Fig. 4B), indicating recent exposure and high prevalence of flu infections in this population. While age was an important contributor to the magnitude of the humoral response towards influenza, sex and CCIw did not seem to play a significant role (Table 3).

**Table 3:** Summary of LMMs to examine the relationship between Influenza strain-specific antibody levels at different sampling periods, adjusting for age, sex, batch and comorbidities

	InfA Croatia		InfA Wisconsin		InfB Austria	
	IgG	IgM	IgG	IgM	IgG	IgM
Sampling period						
Spring 2022	0.7877***	0.4536***	0.3483***	0.2572***	0.4481***	0.3428***
	[0.65, 0.92]	[0.37, 0.54]	[0.24, 0.46]	[0.20, 0.31]	[0.33, 0.57]	[0.28, 0.41]
Autumn 2022	0.3827***	0.0481	− 0.0739	0.023	0.0639	0.0413
	[0.24, 0.53]	[− 0.05, 0.14]	[− 0.19, 0.04]	[− 0.04, 0.08]	[− 0.06, 0.19]	[− 0.03, 0.11]
Spring 2023	1.0365***	0.6329***	0.1526**	0.2068***	0.3787***	0.2819***
	[0.90, 1.17]	[0.54, 0.72]	[0.04, 0.26]	[0.15, 0.26]	[0.26, 0.50]	[0.22, 0.35]
Autumn 2023	0.714***	0.2288***	0.0757	0.1156***	0.2901***	0.1267***
	[0.58, 0.85]	[0.14, 0.32]	[− 0.03, 0.19]	[0.06, 0.17]	[0.17, 0.41]	[0.06, 0.19]
Spring 2024	1.2408***	0.2433***	0.1719**	0.1255***	0.4405***	0.1588***
	[1.10, 1.38]	[0.15, 0.34]	[0.06, 0.28]	[0.07, 0.18]	[0.32, 0.56]	[0.09, 0.23]
Autumn 2024	0.7213***	0.0888	− 0.0234	0.0784**	0.228***	0.0579
	[0.58, 0.86]	[0.00, 0.18]	[− 0.14, 0.09]	[0.02, 0.14]	[0.11, 0.35]	[− 0.01, 0.12
Age	0.0043	0.0268***	0.0067*	0.0077***	− 0.0152***	0.0087***
	[− 0.006, 0.015]	[0.02, 0.03]	[0.0004, 0.0130]	[0.004, 0.012]	[− 0.023, − 0.008]	[0.004, 0.013]
Sex						
Male	0.0341	0.04	0.0542	− 0.0404	0.1192	− 0.0485
	[− 0.13, 0.20]	[− 0.07, 0.15]	[− 0.05, 0.15]	[− 0.11, 0.02]	[− 0.003, 0.241]	[− 0.12, 0.03]
CCIw	0.029	− 0.0107	0.022	− 0.0122	0.0182	− 0.0118
	[− 0.01, 0.07]	[− 0.036, 0.015]	[− 0.002, 0.046]	[− 0.027, 0.003]	[− 0.011, 0.047]	[− 0.030, 0.006]

Estimate [95% CI]

*p < 0.05

**p < 0.01

***p-value < 0.001

A majority of Swedish LTCF residents receive an annual influenza vaccine boost during the last quarter of each year. Therefore, in contrast to RSV, we expected a general and cyclic elevation of influenza-specific antibodies in the LTCF population, and that it would peak following the vaccine season. We estimated the proportion of individuals with at least 2-fold-increase in influenza strain-specific IgG or IgM or both compared to the previous sampling time-point (Fig. 4C). This change in antibody levels was primarily vaccination-driven, but infections cannot be excluded as there was no PCR testing. A strong seasonal trend was observed that peaked each spring, demonstrating a robust vaccine-induced response in this population. The effect was highest in spring 2022 (42.65% InfA H1N1, 55.15% InfA H3N2, 47.8% InfB; Fig. 4C). This was attributed to the relatively low antibody levels before vaccination in autumn 2021 (Fig. 4A, B), perhaps due to lack of exposure to influenza virus during the COVID-19-related visiting restrictions in the Swedish LTCFs. Influenza A was more prevalent than influenza B (Fig. 4D) and despite protection by vaccination, breakthrough infections had occurred, since we observed elevated levels of influenza IgG in autumn, i.e., outside of the annual flu seasons and before vaccination (Fig. 4D).

### Antigen-specific antibody profiles improve mortality prediction

**Fig. 5:**
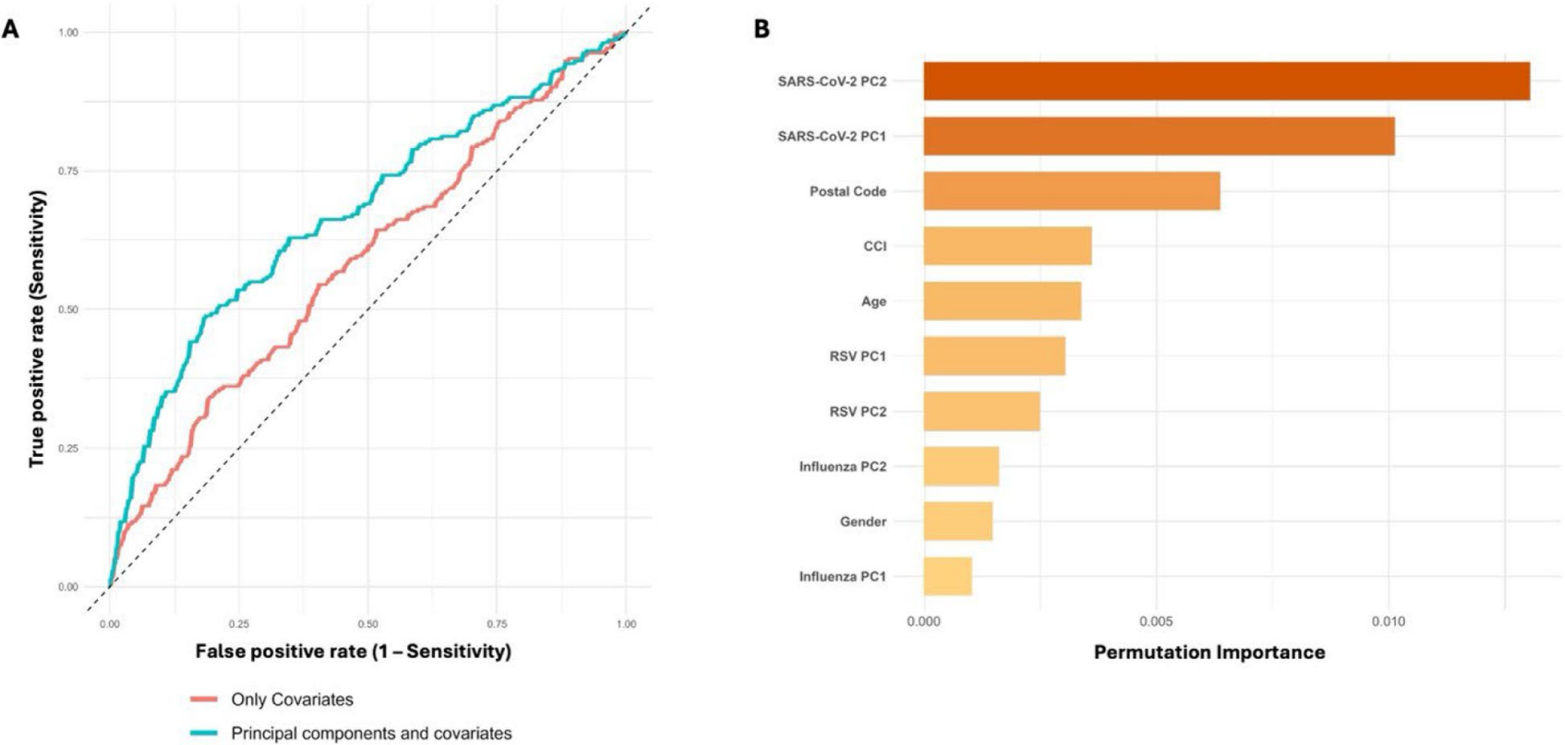
Random forest classifier models to predict mortality.** A** ROC curves comparing the predictive performance of models using only demographic and clinical covariates (red line) versus models including both covariates and antibody principal components (blue line). The combined model showed improved classification accuracy with higher AUC. **B** Permutation-based variable importance scores from the combined model

The overall mortality in the study population was 45% during the study period. We used random forest classifiers to explore whether humoral immune responses against prominent respiratory pathogens contributed to mortality prediction. We compared ROC curves for the models using only demographic and clinical covariates versus the models incorporating both covariates and principal components (PCs) derived from antibody response profiles (Fig. 5A). The inclusion of antibody-derived PCs improved model discrimination, with the AUC increasing from 0.584 for the covariates-only model to 0.672 for the combined model. This improvement in ROC-AUC was statistically significant (DeLong’s test *p* = 0.0015, Bootstrap test *p* = 0.0013). Permutation-based feature importance analysis identified SARS-CoV-2 PCs as the strongest predictors, followed by geographical location (postal code), comorbidity burden (CCIw) and age (Fig. 5B). PCs derived from RSV and influenza responses had moderate to low importance and gender contributed minimally. These findings underscore the importance of respiratory pathogen-specific antibody levels along with demographics and clinical profiles in predicting mortality. Calibration analysis indicated reasonable agreement between predicted and observed mortality at the group level (Additional file 3: S11). The Brier score of 0.226 suggested limited accuracy of individual-level predictions, in line with the model’s moderate discrimination. These findings suggest that respiratory antigen-specific antibody levels, together with demographic and clinical parameters, may provide some additional information for population-level risk stratification, although individual-level prediction remains limited.

### Early post-pandemic RSV exposure and subsequent mortality: exploratory analysis

**Fig. 6:**
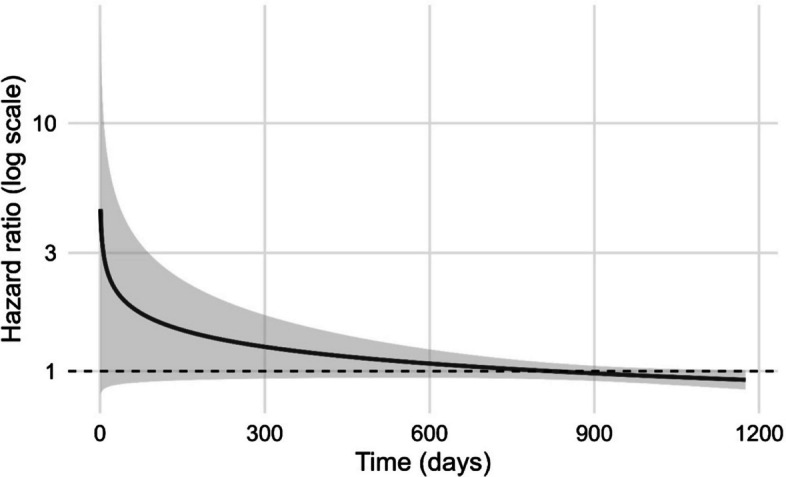
Estimated hazard ratio for pre-F-specific antibodies over time

Since there was very low RSV circulation during 2020–2021 due to public health measures for COVID-19 and there was no vaccine available, we sought to investigate whether RSV exposure during this period of low population immunity was associated with subsequent mortality risk in older adults living in LTCFs. An increase in pre-F-specific IgG levels was used as a proxy for RSV exposure. A baseline Cox proportional hazards model was fitted and adjusted for age, sex, batch and CCIw, accounting for clustering by individual (Additional file 3: S12). RSV pre-F-specific IgG levels were not significantly associated with mortality risk (HR = 0.992, 95% CI 0.922–1.067, *p* = 0.822). In contrast, higher age and higher comorbidity burden were significantly associated with increased mortality risk, as was male sex. Batch effect was not significant. To account for potential changes in antibody effects over time, we then fitted a time-varying Cox model including an interaction between antibody levels and log-transformed time since baseline (Table 4). This model suggested a trend towards an elevated early hazard of death associated with higher RSV pre-F antibody levels at the beginning of the study period (HR = 4.50, 95% CI 0.80–25.20, *p* = 0.087), but this effect attenuated over time (interaction HR = 0.80, 95% CI: 0.62–1.03, *p* = 0.081). Age, male sex and comorbidity burden remained significant predictors of mortality risk, with effect sizes similar to the baseline model. While there was an elevated hazard ratio shortly after baseline, this declined towards the null with increasing follow-up time (Fig. 6).

**Table 4:** Time-varying Cox proportional hazards model

Variable	HR	95% CI	*p*-value
Antibody level	4.500	0.80–25.20	0.087
Antibody level × log(time)	0.799	0.62–1.03	0.081
Age	1.028	1.02–1.04	< 0.001
Male sex	1.223	1.04–1.44	0.013
Batch	1.004	1.00–1.01	0.216
CCIw	1.070	1.03–1.11	< 0.001

To estimate the effect of RSV pre-F antibodies on mortality at the first sampling period when there was low population level immunity, we performed logistic regression of the RSV pre-F IgG levels in autumn 2021 and demographics. The model implied that higher antibody levels were associated with increased odds of one-year mortality (OR = 1.43, 95% CI 1.05–1.97, *p* = 0.024), while age, sex and comorbidities were not significant predictors (Additional file 3: S13). Moreover, these deaths were not all COVID-19-related (Additional file 3: S14), in line with the trends suggested by the Cox proportional hazards and logistic regression models. Taken together, these exploratory analyses suggest trends of a transient association between higher RSV pre-F IgG levels and one-year mortality early in the post-pandemic period, when population-level RSV immunity was low.

## Discussion

Older adults over 65 years of age residing in LTCFs are under-represented in seroprevalence studies in Sweden and worldwide. This study leverages a unique longitudinal sampling platform established during the COVID-19 pandemic and extending through 2024, enabling comprehensive assessment of humoral immunity to respiratory infections in this vulnerable population. By integrating serological data and registry information, we provide a temporal and regional overview of antibody dynamics to RSV, influenza and SARS-CoV-2 spanning autumn 2021 to 2024.

We observed robust and sustained antibody responses to SARS-CoV-2 after the third vaccine dose in autumn 2021, similar to what was previously shown by us and others [23, 33, 34]. The increase in spike-specific IgG and IgM levels in spring 2022 coincided with the relaxation of COVID-19-related restrictions and increased opportunities for natural exposure, suggesting contributions from both vaccination and community transmission. Seasonal stratification showed high and stable IgG levels in both spring and autumn sampling periods throughout 2022 to 2024, indicating sustained immunological memory in this population due to biannual vaccination.

RSV is an important contributor to disease burden in both community-dwelling older adults and those residing in LTCFs [35]. However, most incidence studies in adults rely on cases seeking medical care, thereby missing infections that are mild or asymptomatic. In our study, participants were not selected based on acute respiratory illness, and in the absence of PCR testing or a validated serological threshold for RSV seropositivity, we used fold increases in antibody levels as a proxy for infection or exposure. While a 4-fold increase is conventionally used to indicate recent infection, this threshold may underestimate incidence in longitudinal studies with longer sampling intervals, since pre-existing immunity and antibody waning can mask infection events. For this reason, some experts have recommended a 2-fold increase as a more sensitive population-level indicator [30, 31, 36, 37].

To account for the complexity of repeated exposures and pre-existing immunity in older adults, we compared the estimates of infection burden using 1.5-, 2- and 4-fold increase of RSV pre-F antibodies (Fig. 2C, D). While absolute estimates of RSV burden varied by threshold, the temporal trends and peaks remained consistent, supporting the robustness of our conclusions. Nonetheless, using smaller fold increases may slightly overestimate infection events, while relying only on 4-fold rises could underestimate the true population exposure. We provide a transparent view of both the likely minimum and maximum RSV burden in this cohort by presenting results across all thresholds. Using this comprehensive approach, our data suggest a substantially higher RSV infection burden in Sweden than indicated by PHAS PCR surveillance. This is consistent with findings from a recent U.S. serosurveillance study, which revealed widespread undiagnosed RSV infections, waning immunity and frequent asymptomatic cases across both infected and vaccinated adults [38]. Given that a large proportion of respiratory infections are asymptomatic in older adults who have experienced repeated exposures throughout life [27, 28, 39, 40], our findings likely capture a more complete picture of RSV circulation than case-based surveillance alone. As there are uncertainties in serological thresholds and long sampling intervals, our serological estimates indicate under-ascertainment rather than precise incidence rates. These should be interpreted as indicators of infection burden independent of symptoms or healthcare-seeking behaviour.

RSV-specific antibody levels in our cohort showed marked regional and temporal variation. An increase in IgM levels and G-protein-specific IgG was observed in early 2022, coinciding with the removal of COVID-19-related visiting restrictions in LTCFs. This pattern suggests increased RSV exposure in the context of low population-level immunity. High IgG levels were maintained until late 2022, likely conferring short-term protection reflected in the subsequent decline in IgM levels. By spring 2023, RSV antigen-specific IgG titers had significantly waned, reducing antibody-mediated protection and preceding a surge in new exposures in autumn 2023. This boost in immunity then persisted throughout 2024. Together, these trends highlight the dynamic circulation of RSV in LTCFs and the interplay between infection and immunity.

Even though a majority of the RSV infections may be subclinical, our exploratory survival analysis and logistic regression models suggest that higher levels of RSV pre-F antibodies, interpreted as markers of recent population-level RSV exposure, may be associated with a transiently elevated hazard of death early in follow-up. However, this effect attenuated over time, consistent with the long-term protective role of pre-F-specific neutralising antibodies. The observed associations between RSV pre-F antibody levels and mortality warrant cautious interpretation given the modest effect size, wide confidence interval and *p*-value higher than the conventional threshold. Moreover, residual confounding by frailty, immune competence, healthcare contact, or unmeasured clinical factors cannot be excluded. These results require validation in independent cohorts. The research question is clinically relevant, as the risk could potentially be modified through vaccination and/or monoclonal antibody treatments. The association of pre-F-specific antibody levels with a transiently increased risk of adverse outcomes in this population is presumably a broad effect of low population-level immunity during the pandemic, rather than a direct effect of RSV exposure on mortality.

The differing dynamics of G- and F-specific antibodies in our study indicate circulation of both RSV A and B strains, and we found substantial RSV transmission outside the typical annual season, highlighting the potential value of vaccination in this vulnerable population. Notably, serological evidence revealed a higher RSV burden compared to that reported by PHAS in the general population. A proportion of the differences could be accounted for by the nature of testing as PHAS uses PCR detection of the virus to determine infection rates which only specifically captures active infections and not recent exposure. Moreover, the infection rates determined by PHAS are using samples from individuals who report to the hospital, while older adults in Swedish LTCFs do not typically seek care for respiratory infection symptoms.

Influenza antibody profiles showed recurrent seasonal boosting due to annual vaccination. The decline in antibody levels in autumn 2024 paralleled a drop in infection rates, possibly reflecting cumulative population immunity and/or decreased viral circulation. In addition, data from the population surveillance by PHAS showed a very early influenza season 2023–2024 and a very late influenza season 2024–2025 [41], complementing our results. These dynamics underscore the relevance of ongoing serological monitoring for evaluating vaccine-induced immunity and detecting emerging gaps in protection.

The random forest classifier suggests that humoral immune responses may enhance mortality risk prediction beyond demographic and clinical variables alone. Traditional factors such as age, comorbidity burden and geographical location remained relevant, but SARS-CoV-2 antibody features contributed substantially to model performance. This observation is consistent with differences in antibody levels between individuals who respond robustly to COVID-19 vaccination and those who do not, suggesting that the model may partly capture variation in immune responsiveness. These findings indicate that serological data could provide additional information for refining population-level mortality risk models and support their potential integration into broader public health surveillance strategies. In practice, such models could be used to monitor risk across LTCFs, identify groups at higher aggregate risk and guide infection-prevention measures, rather than to make precise predictions for individual residents.

Several limitations warrant consideration. One caveat in this study is that we did not have information about voluntary uptake of the recently approved RSV vaccines in this cohort. Since the RSV vaccine for older adults was approved in autumn 2023, the sampling periods before that clearly represent infection-driven immune responses, however, in the later time-points, vaccination coverage is still assumed to be very low, if at all, due to it not being subsidised. However, there is no national registry data available to confirm this. With RSV vaccines now recommended in Sweden for older and high-risk adults [42], our findings demonstrate how longitudinal serological surveillance in LTCFs could support targeted immunisation by identifying periods of low population immunity and localised RSV circulation, complementing symptom-based surveillance where clinical detection is limited.

Another limitation is that more than half of the participants contributed only a single sample, limiting longitudinal follow-up. Mortality was higher among those with one sample than those with repeated sampling (53% vs 37%), consistent with greater frailty restricting continued participation. This selective loss to follow-up due to death, declining health, or non-participation may create bias and likely enriches longitudinal analyses for healthier survivors. Accordingly, fold-increase analyses were restricted to those with paired samples and should be interpreted as reflecting exposure dynamics in a subset with sufficient survival and participation. Batch effects were detected and statistically adjusted for but may still introduce residual confounding. Further, the study relied on serological kinetics to infer infection status rather than PCR-confirmed diagnoses. This approach is valid in cohort settings; however, the lack of virological confirmation introduces uncertainty. In addition, we were unable to identify an age-matched negative control with no previous exposure to RSV, SARS-CoV-2 and influenza.

## Conclusions

The study provides important insights into population-level immunity and demonstrates the value of integrating serological and epidemiological data to monitor immunity in older adults in assisted living. Our approach highlights the value of DBS sampling for large-scale surveillance in vulnerable populations and offers critical support for evidence-based vaccine scheduling and preparedness planning as viral transmission patterns evolve in the post-pandemic period. We demonstrate sustained SARS-CoV-2 responses following vaccination, dynamic RSV circulation with marked regional and temporal variation, and seasonal boosting of influenza antibodies. Further, we reported evidence of substantial RSV burden in this population that is underestimated by conventional surveillance. Importantly, we showed that integrating serological profiles with registry data improved mortality risk prediction beyond clinical factors alone. These findings underscore the utility of serological surveillance for guiding vaccination strategies, refining risk models and strengthening preparedness for respiratory infections in frail older adults. Ongoing surveillance and further research into correlates of protection, including mucosal immunity and soluble biomarkers, will be essential to protect those at highest risk.

## Supplementary Information

Supplementary Material 1: S1-S6. S1 – Antigen details. S2 – Participant demographics. S3 – Comparison between baseline characteristics. S4 – Assay validation. S5 – SARS-CoV-2 antibody responses. S6 – Summary of SARS-CoV-2-specific IgG and IgM LMMs.

Supplementary Material 2: S7-S10. S7 – RSV antigen-specific antibody responses. S8—Correlations between RSV antigen-specific IgG and IgM levels. S9 – RSV seroprevalence by region. S10 – Influenza strain-specific antibody responses.

Supplementary Material 3: S11-S14. S11 – Calibration plot of the mortality prediction model. S12 – Baseline Cox proportional hazards model. S13 – Logistic regression. S14 – Mortality over time.

## Data Availability

Data sharing is regulated by the General Data Protection Regulation 2016/679, the Swedish Law on Biobanking and approved ethical permits. The study data comprises personally identifiable and sensitive information that cannot be shared. Data from the deidentified administrative health registry are not freely available due to protection of personal integrity of the participants.

## Abbreviations

ANOVA: Analysis of variance
AUC: Area under curve
CCI: Charlson Comorbidity Index
CCIw: CCI weighted score
CI: Confidence interval
DBS: Dried blood spots
EMM: Estimated marginal mean
F: Fusion protein
G: Glycoprotein
HA: Hemagglutinin
HR: Hazard ratio
LMM: Linear mixed-effects model
LTCF: Long-term care facility
MFI: Median fluorescence intensity
OR: Odds ratio
PBS-T: Phosphate Buffered Saline with 0.05% Tween-20
PBS-TBN: PBS-T with 0.1% bovine serum albumin and 0.05% sodium azide
PC: Principal component
PCA: Principal Component Analysis
PHAS: The Public Health Agency of Sweden
post-F: RSV post-fusion protein
pre-F: RSV pre-fusion protein
ROC: Receiver operating characteristic curve
RSV: Respiratory syncytial virus
RT: Room temperature
SARS-CoV-2: Severe acute respiratory syndrome Coronavirus 2
