# Supplementary material for: Serological evidence of substantial respiratory syncytial virus infection burden among older adults residing in Swedish long-term care facilities
Source: BMC Med. 2026 Feb 24;24:134. doi: 10.1186/s12916-026-04700-7 (PMC12955034; doi:10.1186/s12916-026-04700-7)
Supplement: Supplementary file 1 — Supplementary Material 1: S1-S6. S1 – Antigen details. S2 – Participant demographics. S3 – Comparison between baseline characteristics. S4 – Assay validation. S5 – SARS-CoV-2 antibody responses. S6 – Summary of SARS-CoV-2-specific IgG and IgM LMMs. [file 12916_2026_4700_MOESM1_ESM.docx]

**S1. Antigen details.**

| **Bead region** | **Antigens** | **Concentration**  **(per million beads)** |
| --- | --- | --- |
| 27 | RSV pre-F (produced in-house) | 10 μg |
| 34 | SARS-CoV-2 (Wuhan) spike (produced in-house) | 5 μg |
| 35 | Influenza A H3N2 (A/Croatia/10136RV/2023) Hemagglutinin (Cat# H32-V52H4, ACRO Biosystems) | 3 μg |
| 36 | RSV post-F (produced in-house) | 5 μg |
| 44 | RSV (subtype A) G protein (Cat#40041-V08H, Sino Biological) | 5 μg |
| 46 | Influenza A H1N1 (A/Wisconsin/67/2022) Hemagglutinin (Cat#40940-V08B, Sino Biological) | 5 μg |
| 52 | Influenza B (B/Austria/1359417/2021) Hemagglutinin (Cat#40862-V08B, Sino Biological) | 3 μg |

**S2. Participant demographics.**

A. Geographical locations of residential LTCFs in two metropolitan areas (Stockholm and Skåne; blue colour) and three less densely populated regions (Västerbotten, Jämtland-Härjedalen and Örebro; red colour); B. Number of participants (n=1622) assessed from each region, grouped by sex; C. Age distribution of study participants (n=1622) stratified by sex.


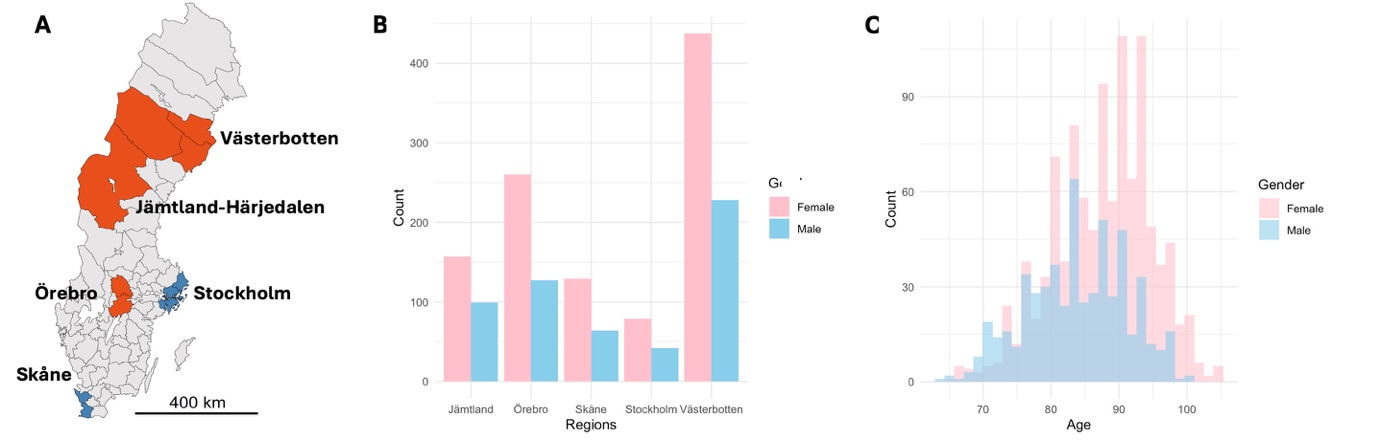


**S3. Baseline characteristics.** Comparison between participants contributing a single sample and those with longitudinal follow-up (>1 sample).

|  | **Overall**  N = 1,622*^1^* | **1 sample**  N = 881*^1^* | **>1 sample**  N = 741*^1^* | **p-value***^2^* |
| --- | --- | --- | --- | --- |
| **Age** | 87 (81, 92) | 87 (81, 92) | 86 (81, 92) | 0.094 |
| **Sex** |  |  |  | 0.179 |
| Female | 1,062 (65%) | 564 (64%) | 498 (67%) |  |
| Male | 560 (35%) | 317 (36%) | 243 (33%) |  |
| **Myocardial infarction** | 211 (13%) | 123 (14%) | 88 (12%) | 0.214 |
| **Congestive heart failure** | 268 (17%) | 150 (17%) | 118 (16%) | 0.552 |
| **Peripheral vascular disease** | 119 (7.3%) | 66 (7.5%) | 53 (7.2%) | 0.794 |
| **Cerebrovascular disease** | 452 (28%) | 246 (28%) | 206 (28%) | 0.956 |
| **Chronic obstructive pulmonary disease** | 98 (6.0%) | 63 (7.2%) | 35 (4.7%) | 0.041 |
| **Chronic other pulmonary disease** | 118 (7.3%) | 64 (7.3%) | 54 (7.3%) | 0.986 |
| **Rheumatic disease** | 118 (7.3%) | 61 (6.9%) | 57 (7.7%) | 0.553 |
| **Dementia** | 839 (52%) | 463 (53%) | 376 (51%) | 0.467 |
| **Hemiplegia** | 153 (9.4%) | 80 (9.1%) | 73 (9.9%) | 0.597 |
| **Diabetes without chronic complication** | 274 (17%) | 159 (18%) | 115 (16%) | 0.176 |
| **Diabetes with chronic complication** | 113 (7.0%) | 62 (7.0%) | 51 (6.9%) | 0.903 |
| **Renal disease** | 89 (5.5%) | 53 (6.0%) | 36 (4.9%) | 0.308 |
| **Mild liver disease** | 14 (0.9%) | 6 (0.7%) | 8 (1.1%) | 0.387 |
| **Ascites** | 6 (0.4%) | 5 (0.6%) | 1 (0.1%) | 0.228 |
| **Severe liver disease** | 2 (0.1%) | 1 (0.1%) | 1 (0.1%) | >0.999 |
| **Peptic ulcer disease** | 73 (4.5%) | 36 (4.1%) | 37 (5.0%) | 0.380 |
| **Malignancy** | 226 (14%) | 131 (15%) | 95 (13%) | 0.235 |
| **Metastatic solid tumor** | 30 (1.8%) | 18 (2.0%) | 12 (1.6%) | 0.528 |
| **Aids** | 1 (<0.1%) | 1 (0.1%) | 0 (0%) | >0.999 |
| **CCIunw** | 2.00 (1.00, 3.00) | 2.00 (1.00, 3.00) | 2.00 (1.00, 3.00) | 0.046 |
| **CCIw** | 2.00 (1.00, 3.00) | 2.00 (1.00, 3.00) | 2.00 (1.00, 3.00) | 0.048 |
| **Death during study period** |  |  |  | <0.001 |
| No | 888 (55%) | 418 (47%) | 470 (63%) |  |
| Yes | 734 (45%) | 463 (53%) | 271 (37%) |  |
| *^1^*Median (Q1, Q3); n (%) | | | | |
| *^2^*Wilcoxon rank sum test; Pearson's Chi-squared test; Fisher's exact test | | | | |
| Abbreviations: n, number of patients; IQR, interquartile range. | | | | |

**S4. Assay validation.** SARS-CoV-2 spike-specific IgG levels compared with (A) measurements from an alternate serological assay (MSD, USA) and (B) paired serum samples.

**
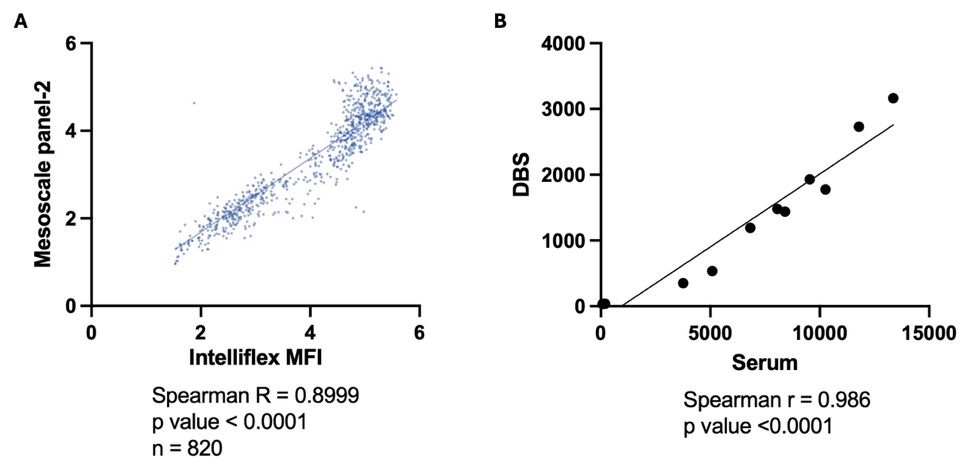
**

**S5. SARS-CoV-2-specific antibody responses following vaccination.** SARS-CoV-2 (Wuhan) spike-specific IgG (A) and IgM (B) levels over time. The data points represent Mean ± SEM and statistical significance is shown relative to September 2021; ***p < 0.001

**
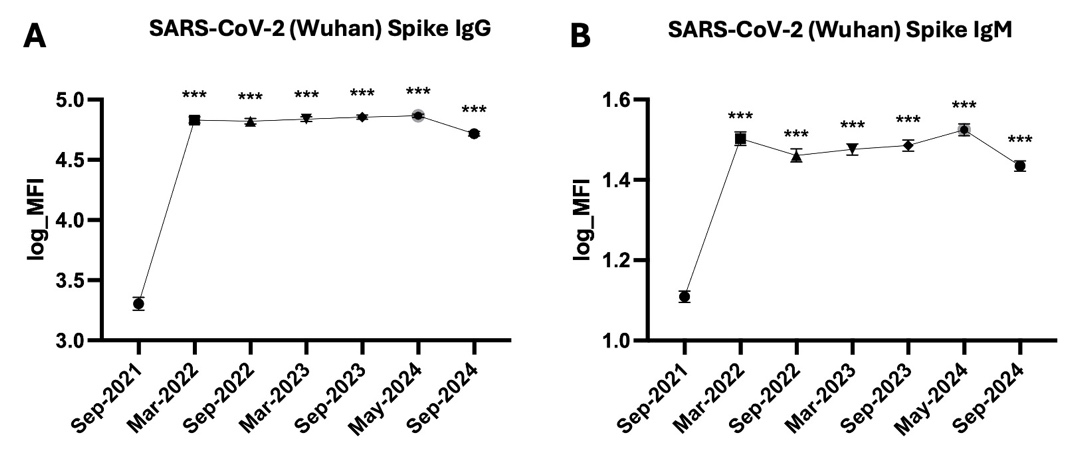
**

S6. Summary of LMMs showing the relationship between SARS-CoV-2 (Wuhan) spike-specific antibody levels at different sampling periods, adjusted for age, sex, batch and comorbidities.

|  | **IgG** | **IgM** |
| --- | --- | --- |
| **Sampling period** |  |  |
| Spring 2022 | 3.3541*** | 0.8244*** |
|  | [3.20, 3.51] | [0.75, 0.90] |
| Autumn 2022 | 3.365*** | 0.7323*** |
|  | [3.20, 3.53] | [0.65, 0.81] |
| Spring 2023 | 3.3506*** | 0.7418*** |
|  | [3.19, 3.51] | [0.67, 0.82] |
| Autumn 2023 | 3.4444*** | 0.7843*** |
|  | [3.29, 3.60] | [0.71, 0.86] |
| Spring 2024 | 3.4804*** | 0.8627*** |
|  | [3.32, 3.64] | [0.79, 0.94] |
| Autumn 2024 | 3.1402*** | 0.6698*** |
|  | [2.98, 3.30] | [0.59, 0.75] |
| **Age** | −0.0005 | 0.0065** |
|  | [-0.008, 0.007] | [0.002, 0.011] |
| **Sex** |  |  |
| Male | 0.1207* | −0.0677 |
|  | [0.006, 0.236] | [-0.1355, 0.0001] |
| **CCIw** | −0.0269 | −0.0054 |
|  | [-0.0541, 0.0003] | [-0.021, 0.011] |

Estimate [95% CI]; *p < 0.05; **p < 0.01; ***p value < 0.001
