# Supplementary material for: Serological evidence of substantial respiratory syncytial virus infection burden among older adults residing in Swedish long-term care facilities
Source: BMC Med. 2026 Feb 24;24:134. doi: 10.1186/s12916-026-04700-7 (PMC12955034; doi:10.1186/s12916-026-04700-7)
Supplement: Supplementary file 2 — Supplementary Material 2: S7-S10. S7 – RSV antigen-specific antibody responses. S8—Correlations between RSV antigen-specific IgG and IgM levels. S9 – RSV seroprevalence by region. S10 – Influenza strain-specific antibody responses. [file 12916_2026_4700_MOESM2_ESM.docx]

**S7. RSV antigen-specific antibody responses.** RSV pre-F-, post-F- and G-specific (A) IgG and (B) IgM levels over time. The data points represent Mean ± SEM and statistical significance is shown relative to September 2021; *p < 0.05, **p < 0.01, ***p < 0.001

**
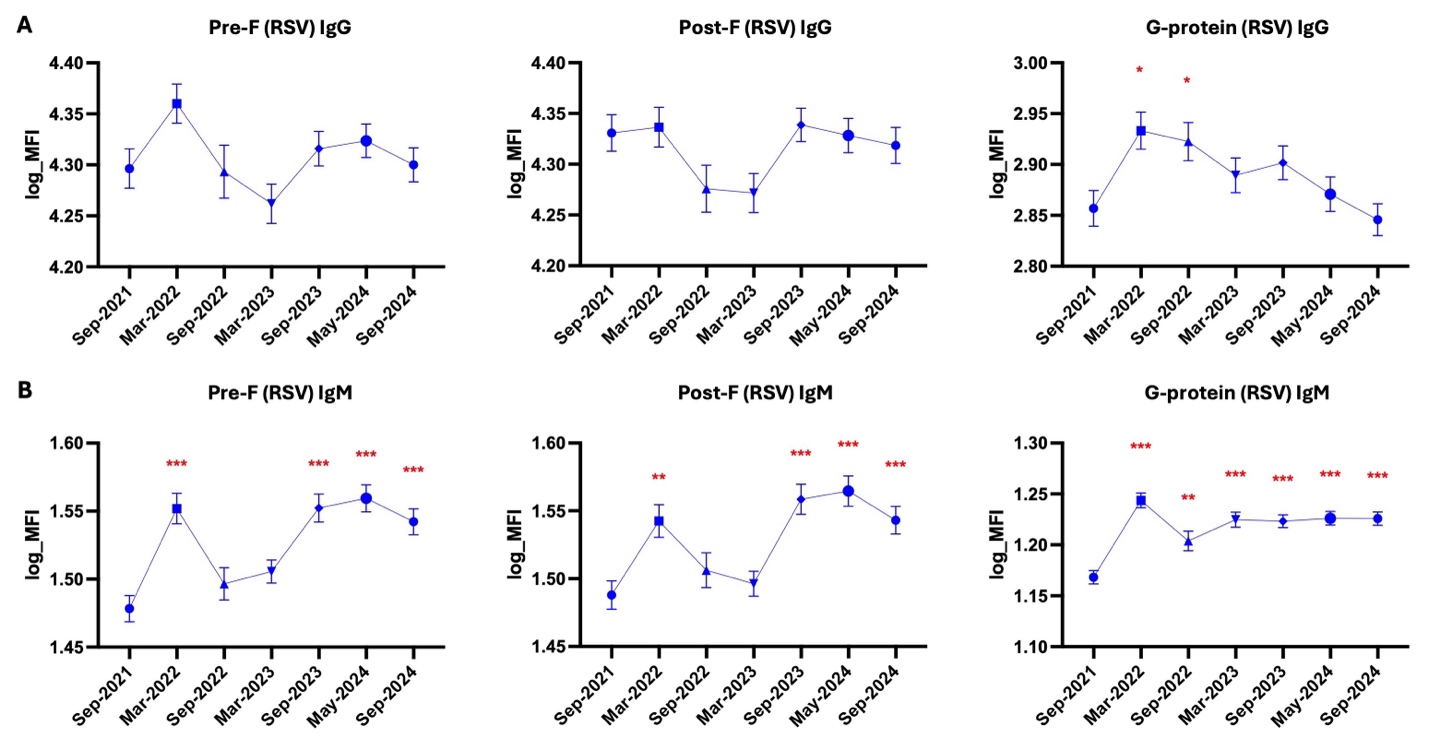
**

S8. Spearman correlations between RSV antigen-specific IgG and IgM levels at different sampling periods during the study.

**
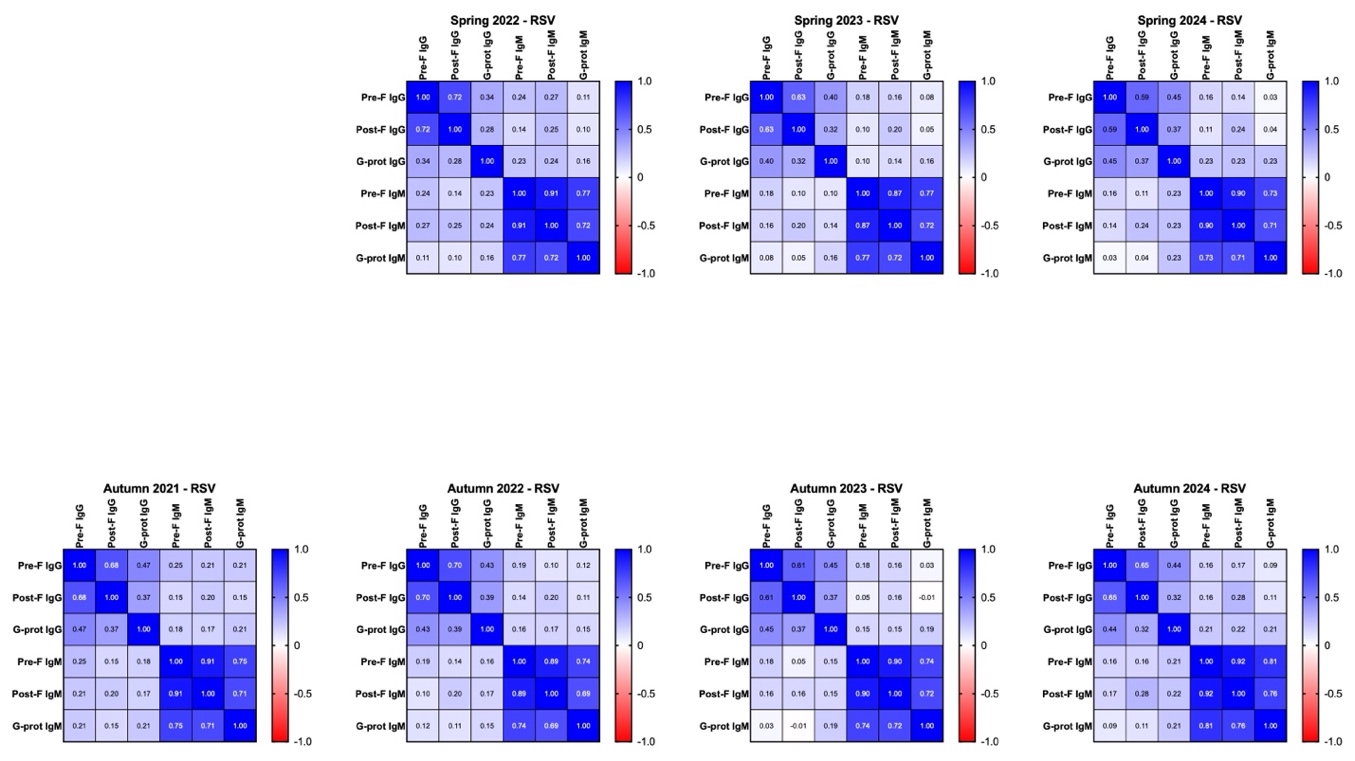
**

**S9. RSV seroprevalence by region.** RSV pre-F-, post-F- and G-specific (A) IgG and (B) IgM levels over time in five regions of Sweden. The data points represent Mean ± SEM

**
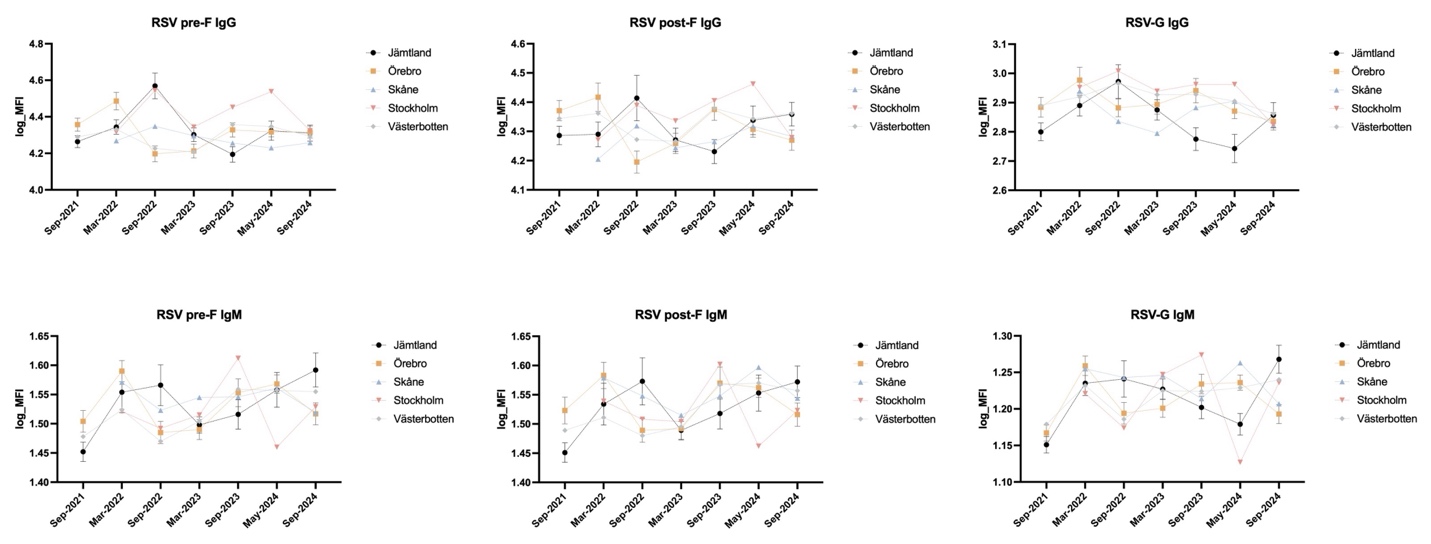
**

**S10. Influenza strain-specific antibody responses.** Influenza strain-specific (A) IgG and (B) IgM levels over time. The data points represent Mean ± SEM and statistical significance is shown relative to September 2021; *p < 0.05, **p < 0.01, ***p < 0.001

**
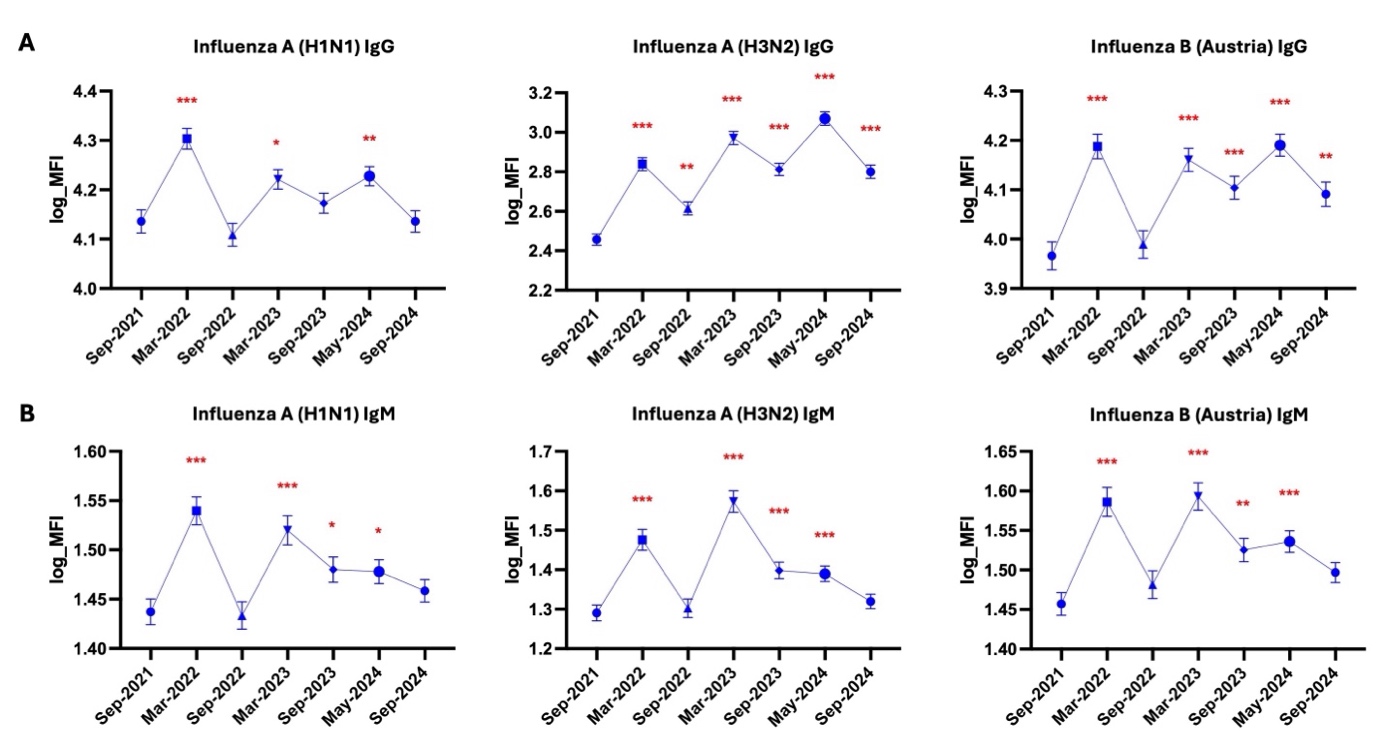
**
