# Supplementary material for: Serological evidence of substantial respiratory syncytial virus infection burden among older adults residing in Swedish long-term care facilities
Source: BMC Med. 2026 Feb 24;24:134. doi: 10.1186/s12916-026-04700-7 (PMC12955034; doi:10.1186/s12916-026-04700-7)
Supplement: Supplementary file 3 — Supplementary Material 3: S11-S14. S11 – Calibration plot of the mortality prediction model. S12 – Baseline Cox proportional hazards model. S13 – Logistic regression. S14 – Mortality over time. [file 12916_2026_4700_MOESM3_ESM.docx]

**S11. Calibration plot of the mortality prediction model.**

**
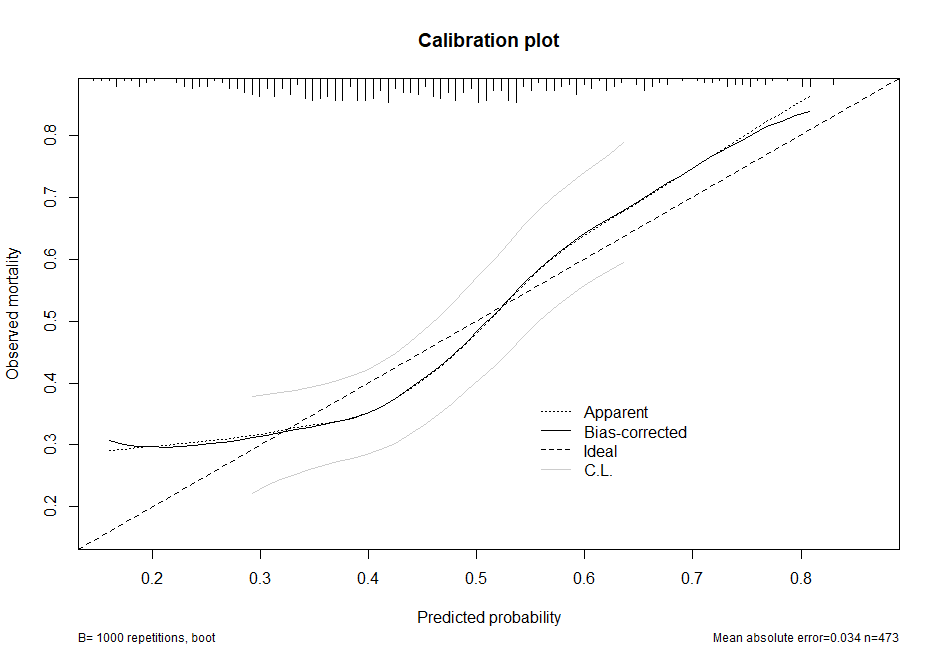
**

**S12. Baseline Cox proportional hazards model**

| **Variable** | **HR** | **95% CI** | **p-value** |
| --- | --- | --- | --- |
| Antibody level | 0.992 | 0.922–1.067 | 0.822 |
| Age | 1.028 | 1.017–1.039 | <0.001 |
| Male sex | 1.226 | 1.045–1.439 | 0.012 |
| Batch | 1.005 | 0.998–1.012 | 0.177 |
| CCIw | 1.069 | 1.032–1.106 | <0.001 |

**S13. Logistic regression results**

| **Coefficients** | **OR** | **95% CI** | **p-value** |
| --- | --- | --- | --- |
| log_MFI | 1.43 | 1.05–1.97 | 0.024* |
| Age | 1.01 | 0.98–1.04 | 0.624 |
| Gender (Male) | 1.21 | 0.71–2.05 | 0.482 |
| Batch | 1.02 | 0.99–1.04 | 0.250 |
| CCIw | 0.99 | 0.88–1.11 | 0.884 |

**S14. Mortality over time.** Comparison of deaths within 30 days of SARS-CoV-2 infection and all other deaths

**
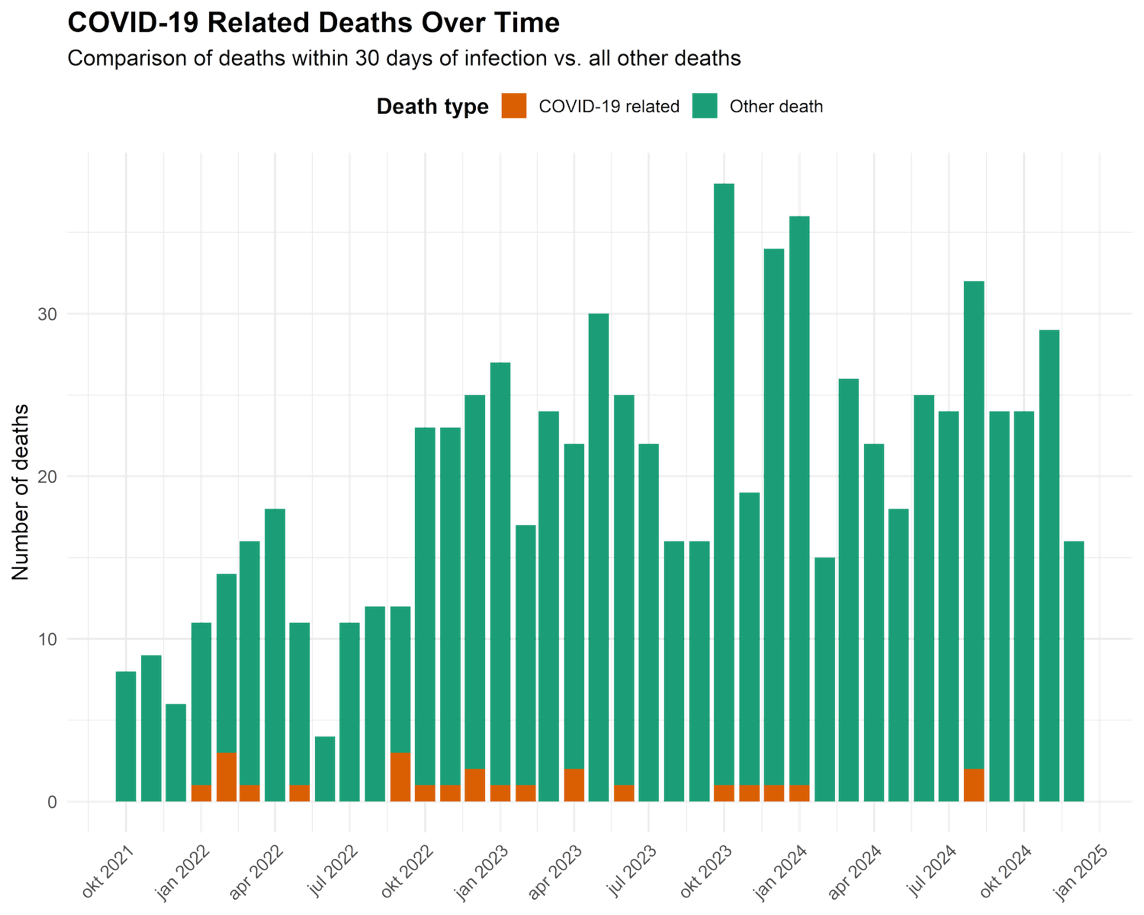
**
